# A self-healing radiopaque hyaluronic acid hydrogel as a new injectable biomaterial for precision medicine in osteoarthritis

**DOI:** 10.7150/thno.104551

**Published:** 2025-03-10

**Authors:** Moustoifa Said, Clément Tavakoli, Chloé Dumot, Karine Toupet, Cécile Olivier, Alexia Gilles, Marie Maumus, Yuxi Clara Dong, Nora Collomb, Céline Auxenfans, Anaïck Moisan, Bertrand Favier, Benoit Chovelon, Emmanuel Luc Barbier, David Peter Cormode, Emmanuel Brun, Hélène Elleaume, Marlène Wiart, Olivier Detante, Claire Rome, Danièle Noël, Rachel Auzély-Velty

**Affiliations:** 1 Univ. Grenoble Alpes, Centre de Recherches sur les Macromolécules Végétales (CERMAV-CNRS), 38041 Grenoble, France.; 2 Univ. Grenoble Alpes, Inserm, U1216, Grenoble Institut Neurosciences, 38000 Grenoble, France.; 3 Univ. Lyon 1, Inserm U1060, CarMeN Laboratory, 69600 Oullins, France.; 4 Univ. Grenoble Alpes, Inserm, UA7 Strobe, 38000 Grenoble, France.; 5 Hospices Civils de Lyon, 69677 Bron, France.; 6 IRMB, Univ. Montpellier, INSERM, CHU Montpellier, 34295 Montpellier, France.; 7 Department of Radiology and Department of Bioengineering, University of Pennsylvania, Philadelphia, Pennsylvania 19104, United States.; 8 Hôpital Edouard Herriot, 69003 Lyon, France.; 9 Cell Therapy and Engineering Unit, EFS Rhône Alpes, 38330 Saint Ismier, France.; 10 Univ. Grenoble Alpes, Translational Innovation in Medicine & Complexity, UMR552, 38700 La Tronche, France.; 11 Univ. Grenoble-Alpes, Département de Pharmacochimie Moléculaire UMR 5063, 38400 Grenoble, France.; 12 CHU de Grenoble-Alpes, Institut de Biologie et Pathologie, 38700 La Tronche, France.; 13 CHU Grenoble Alpes, Stroke Unit, Department of Neurology, 38043 Grenoble, France.

**Keywords:** Injectable hydrogel, hyaluronic acid, viscosupplementation, X-ray, Iodine

## Abstract

**Rationale:** Osteoarthritis (OA) is a degenerative disease affecting cartilage, synovium and bone, that is a major cause of pain and disability. Intra-articular injection of hyaluronic acid (HA) derivatives, also known as viscosupplementation (VS), is a common treatment for the symptomatic management of knee OA. Despite its widespread use, the magnitude of the clinical benefit of VS remains controversial, with conflicting results due to methodological differences and possible differences in efficacy between products related to remanence and rheological properties.

**Methods:** Here, to create an effective HA-based treatment, an injectable self-healing HA hydrogel with long-persistent radiopacity is formed by tethering a clinical iodine contrast agent to HA. The labeling conditions are tuned to obtain sufficient X-ray signal without altering the biocompatibility, rheological and injectability properties of the hydrogel.

**Results:** The iodine labeling enabled to monitor not only delivery of the hydrogel but also its retention in mouse knees up to 5 weeks post-administration using synchrotron K-edge subtraction-computed tomography. We further demonstrated that the unique properties of this hydrogel enable creation of a transient HA network *in vivo* that attenuates OA progression in a mouse model of OA. Moreover, our data showed that the rate of HA-I disappearance appears to predict treatment response, likely because a rapid elimination serves as an indirect indicator of *in situ* inflammation.

**Conclusion:** Collectively, these results show that our radiopaque HA-I hydrogel holds significant promise for improving patient management in the treatment of OA before clinical symptoms worsen. Its capacity for *in vivo* tracking over time allows for personalized treatment schedules based on observed retention and therapeutic effect. As a result, this theranostic hydrogel emerges as a strong candidate for precision medicine in OA.

## Introduction

Osteoarthritis is the most common form of arthritis and one of the leading causes of disability. This degenerative and progressive joint disease affects almost 10% of the worldwide population and 6% of the European population, resulting in tremendous individual and socio-economic burden 1. The disease occurs more commonly in elderly patients (over 60 years old) but can also affect younger people or working adults 2. OA is characterized by the damage or breakdown of articular cartilage and subchondral bone, along with alterations in the synovial membrane. The knee is one of the most commonly affected joints, accounting for 60.6 % of all OA cases in 2019 3. There is currently no curative treatment for OA. Current treatment modalities include lifestyle changes (exercise, weight loss), pharmacological therapies, and joint replacement surgeries 4-6. Pharmacologic therapies such as paracetamol, non-steroidal anti-inflammatory drugs, and opioids show efficacy in pain relief but are frequently associated with adverse events 7-9. Intra-articular injection of hyaluronic acid formulations, referred to as viscosupplementation (VS), is a significant next step for patients who have failed to respond to non-surgical treatment options 10. As initially pointed out by Balazs and Denlinger 11, the primary role of HA-based VS is to restore the rheological features of synovial fluid (SF). HA, a major component of SF, contributes substantially to its viscoelastic properties, giving the joint excellent lubrication performance and wear resistance 12-14. This viscoelastic behavior, which is directly linked to both concentration and molar mass of HA, allows the temporary HA network formed by chain entanglements to adapt to the applied mechanical stress 15, 16. In OA, the reduction in the concentration and molar mass of endogenous HA greatly alters the SF properties, causing cartilage damage and worsening OA symptoms 17, 18. Nevertheless, VS effect is not fully clarified due to the multifunctional biochemical role played by HA in joint synovial tissue such as regulation of joint repair through effects on chondrocyte growth and metabolism, promotion of endogenous HA production and various anti-inflammatory effects 19, 20.

Currently, there are several commercially available HA formulations for VS, which differ in HA molar mass, concentration, source (avian or bio-fermentative origin), molecular structure (linear or crosslinked HA) and injected volume. Although the beneficial effects of HA-based VS have been well documented, controversies exist regarding their clinical effectiveness 21, 22. There are several possible explanations for their variable effect on OA patients. Discrepancies may originate from differences in recommended dosing regimens (single or multiple injections), outcome measures, but also differences of efficacy between the HA products. Recommended dosing regimens vary according to the assumed residence time of the HA product into the joint. Indeed, when injected into the joint, HA is rapidly degraded, limiting the residence time from few days for linear molecules to few weeks for cross-linked HA 23-25. Therefore, crosslinked HA products (hydrogels) are receiving increasing attention 22. Compared to other biomacromolecules used to develop injectable hydrogels for OA treatment, HA offers a distinct biological advantage as a primary component of synovial fluid and cartilage. Moreover, HA is widely used in clinical practice, indicating its safety 26, 27. However, the different cross-linking techniques might lead to different levels of effectiveness 28. Moreover, albeit at low incidence, adverse events (pseudoseptic reactions) have been reported with the use of covalently crosslinked HA products 22, 29.

Thus, the ideal HA hydrogel candidate for intra-articular injection therapy in the treatment of OA has yet to be defined. This calls for the study and understanding of the retention and behavior of HA networks in the joint over time using non-invasive imaging tools to link the *in vivo* hydrogel content with the therapeutic effect. The use of imaging for hydrogel delivery monitoring is also key to optimize the chances of successful treatments. Several clinical studies have demonstrated the positive effect of image-guided HA injections on efficacy of VS 30, 31. Common imaging modalities to guide HA injections include ultrasound or X-ray fluoroscopy 32. While both modalities allow for verification of needle placement for injection into the joint space, the latter is the only one that currently enables to see how the injectate spreads through contrast agent injection that affords transient contrast enhancement. X-ray CT imaging is also based on the attenuation of X-rays and allows to visualize three-dimensional (3D) morphology of implanted biomaterials. Meanwhile, X-ray CT imaging has excellent accuracy in assessing bony changes in OA 33, and is more cost-effective and less time-consuming than MRI 34. Moreover, recent technological advances such as dual-energy CT (DECT) and spectral photon-counting CT (SPCCT), which allow to differentiate materials of different effective atomic numbers, have provided added value for evaluating subjects with OA 33, 35, 36. This feature makes these imaging modalities attractive tools to both track HA hydrogels and analyze skeletal changes in the OA knee. However, specific labeling with an X-ray contrast agent is required to detect hydrogels in the joint space. One conventional approach making hydrogels radiopaque is to physically incorporate contrast agents within the polymer network. However, this method does not permit longitudinal monitoring of the hydrogel *in vivo* due to the rapid leakage of contrast agents from the matrix 37, 38.

**Figure 1:**
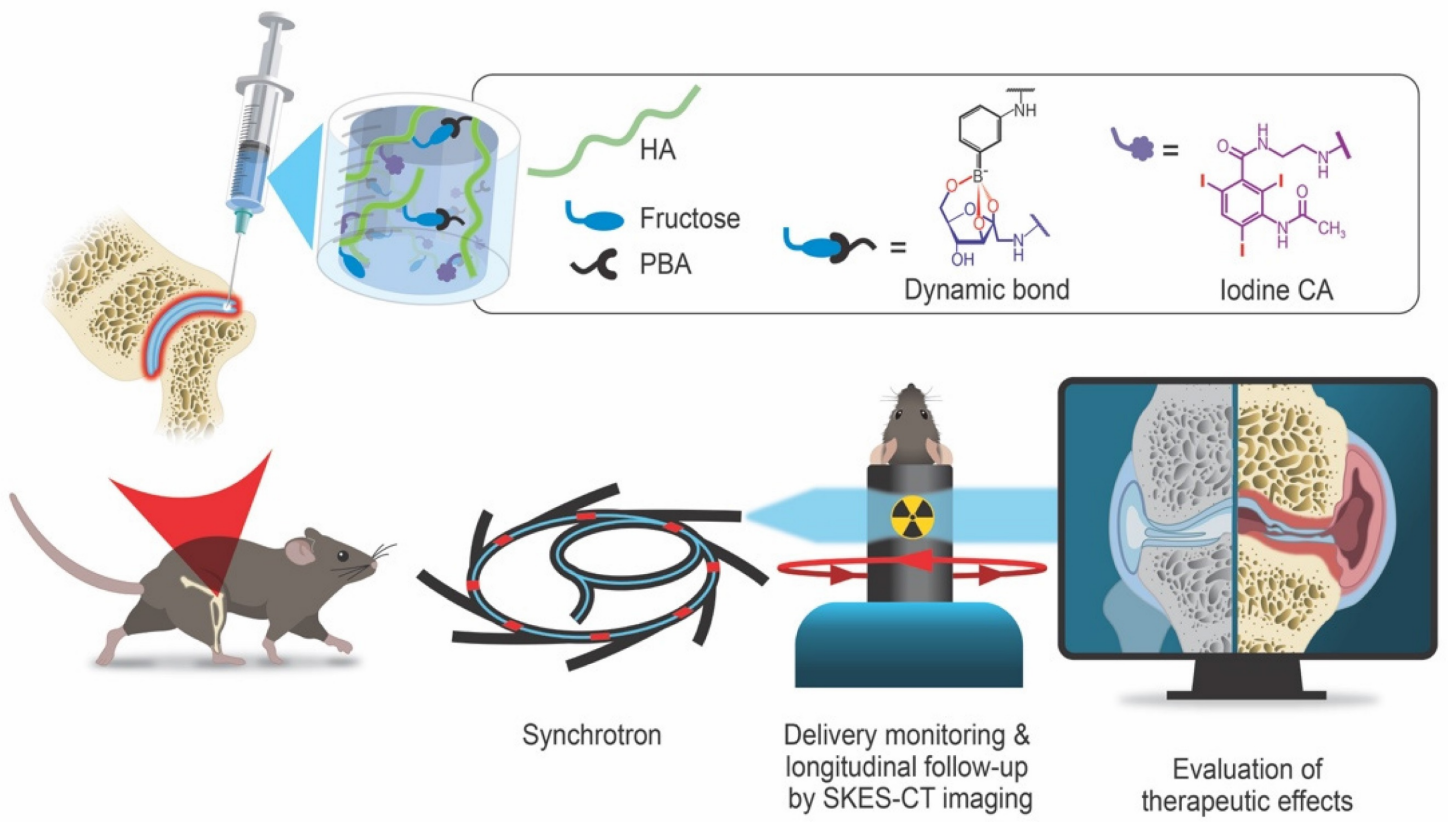
Schematic illustration of the radiopaque and self-healing hyaluronic acid (HA) hydrogel for intra-articular injection in OA. The dynamic cross-links based on boronate ester bonds in the hydrogel network makes it injectable and capable of self-healing almost instantly. The iodine contrast agent (CA) labeling enables monitoring of hydrogel delivery and retention in the knee joint in a mouse model of OA up to 5 weeks post-administration using synchrotron K-edge subtraction computed tomography (SKES-CT). Therapeutic effects are evaluated post-mortem using biological analyses of cartilage and bone degradation.

To the best of our knowledge, no HA hydrogel with strong and long-acting radiopacity for intra-articular injection has been reported for the treatment of OA so far. In this work, we designed and characterized a new iodine-labeled injectable self-healing HA (“HA-I”) hydrogel with stable radiopacity as a potential theranostic candidate in OA. This HA network is crosslinked by dynamic covalent bonds (boronate ester bonds, see **Figure 1**), and can be formulated under mild conditions by simply mixing two solutions of HA partners (one modified with phenylboronic acid (PBA) and the other, functionalized with a fructose derivative (Fru)) in physiological conditions (**Figure 1**). The dynamic cross-links allow the HA network to be extruded under application of shear (needle injection), and rapidly recover the gel state once injection shear is removed 39-42. This self-healing ability not only ensures local hydrogel confinement, but also enables mechanical adaptability that is conducive to maintaining lubrication and joint movement 43. We show that hydrogel labeling through the covalent grafting of a clinical iodine-based contrast agent (i.e. 3-acetamido-2,4,6-triiodobenzoic acid, AcTIB) onto HA does not alter hydrogel biocompatibility, nor its rheological and injectability properties. We further show that it allows its visualization *in vivo* for up to 5 weeks by imaging with synchrotron K-edge subtraction CT (SKES-CT) in the mouse knee. This cutting-edge technology was chosen as a pre-clinical equivalent to clinical SPCCT allowing to reach the high spatial resolution needed to image the mouse knee 44. The unique properties of this hydrogel enable easy administration through needle injection and the creation of a transient HA network *in vivo* that attenuates osteoarthritis progression in a mouse model of OA.

## Results

### Synthesis and characterization of the iodine-labeled HA hydrogel precursors

The preparation of the iodine-labeled injectable HA hydrogel formulation required first the synthesis of the two HA hydrogel precursors, HA-TIB-Fru and HA-TIB-PBA, each labeled with a derivative of a clinical iodine-based contrast agent (AcTIB). Because of the very strong hydrophobicity of AcTIB moieties, the macromolecular parameters of the HA gel precursors (molar mass and degree of substitution (DS, average number of substituting groups per HA disaccharide unit)) were carefully chosen to ensure their solubility in physiological conditions, and to obtain a hydrogel that shows appropriate rheological properties and easy injectability. We previously demonstrated injectability of the non-labeled HA-PBA/HA-Fructose hydrogel prepared from HA derivatives with DS of 0.15 and a weight-average molar mass (M_w_) of 360 kg/mol 45. Therefore, a HA sample with a similar molar mass (M_w_ = 390 kg/mol) was used to prepare the HA-TIB-Fru derivative but for the synthesis of HA-TIB-PBA, an initial HA sample with a lower molar mass (M_w_ = 120 kg/mol) was selected to compensate for the increase in viscosity caused by both hydrophobic AcTIB and PBA moieties attached to the HA backbone.

**Figure 2:**
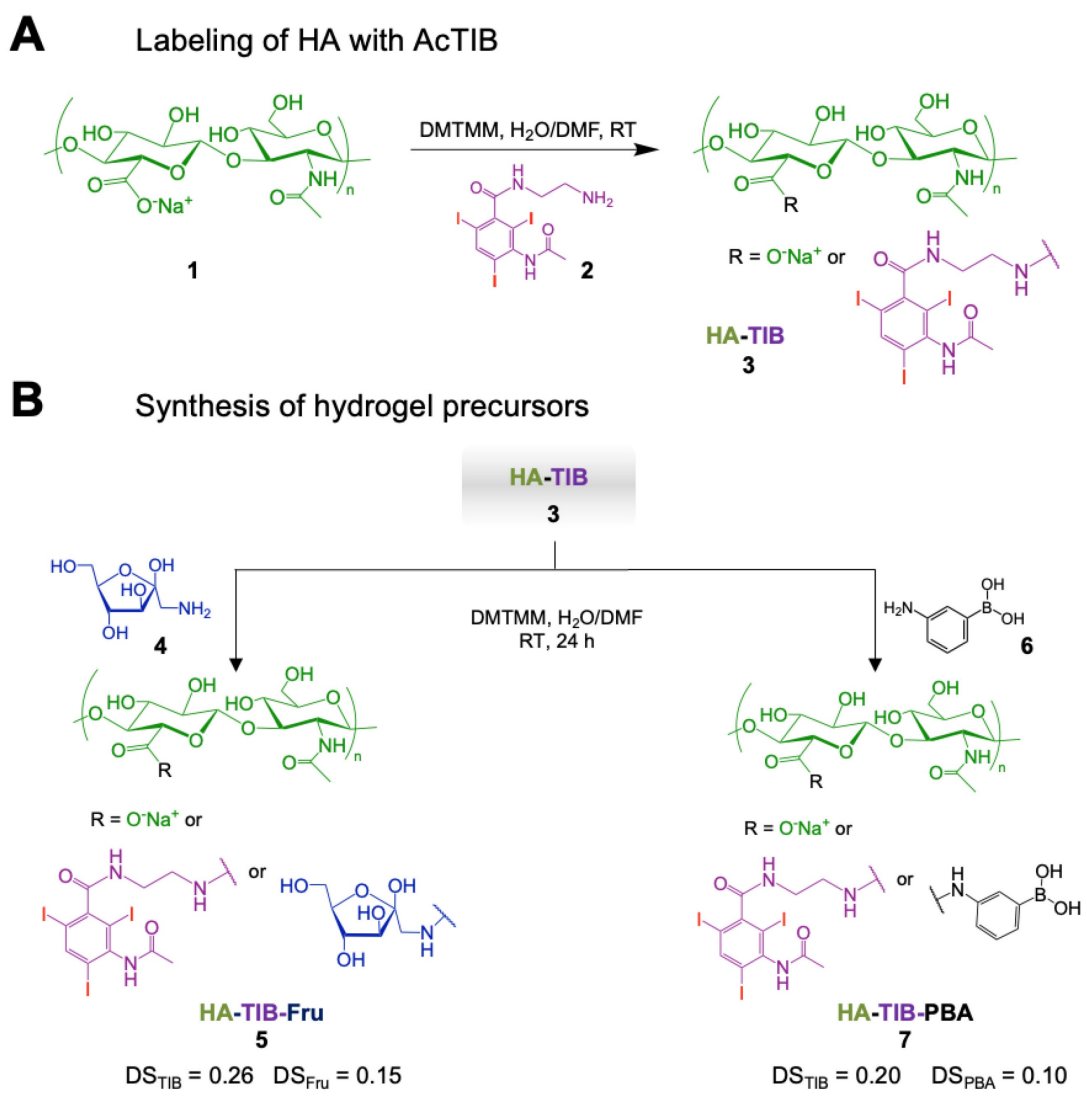
Synthesis of the iodine-labeled HA gel precursors. A) Modification of hyaluronic acid **1** with an iodine-based contrast agent (AcTIB-NH_2_ **2**), affording HA-TIB **3**. B) Grafting of either fructosamine **4** or 3-aminophenylboronic acid **6** on HA-TIB to obtain HA-TIB-Fru **5** and HA-TIB-PBA **7**.

The initial HA were first modified with AcTIB-NH_2_ by an amide coupling reaction using 4-(4,6-dimethoxy-1,3,5-triazin-2-yl)-4-methylmorpholinium chloride (DMTMM) as a coupling agent and, DMTMM/HA and AcTIB-NH_2_/HA molar ratios of 0.36 and 0.60, respectively, to target DS of the HA conjugates of ~ 0.2-0.3 (**Figure 2A**).

AcTIB-NH_2_ was synthesized via amide linkage between* N*-Boc-ethylenediamine and AcTIB followed by removal of the *N*-Boc protecting groups (**Figure S1 and S2**).

Then, HA-TIB was reacted with fructosamine or APBA using DMTMM for amide bond formation 45 (**Figure 2B**). For the synthesis of HA-TIB-Fru **5**, the DMTMM/HA and amine/HA molar ratios were fixed to 1 and 0.15, respectively, to obtain a DS_Fru_ of 0.15. Regarding that of HA-TIB-PBA **7**, the amine/HA molar ratio was decreased to 0.1 to target a DS_PBA_ of 0.1 in order to maintain good water-solubility of the final HA conjugate. The chemical structures of the HA-TIB-Fru and HA-TIB-PBA derivatives were confirmed by ^1^H NMR spectroscopy (**Figure S3 and Figure S4**). Digital integration of the NMR spectra also allowed to assess their DS (DS_TIB_ = 0.26 and DS_Fru_ = 0.15 for HA-TIB-Fru, and DS_TIB_ = 0.20 and DS_PBA_ = 0.10 for HA-TIB-PBA).

The HA compounds showed low toxicity to human adipose-derived stromal cells (hASCs), as assessed by a MTT assay including incubation of HA-PBA, HA-Fru, HA-TIB-PBA and HA-TIB-Fru with hASCs for 72 h at 37° C. hASCs were used because they are an abundant and accessible source of adult stem/stromal cells with multipotent properties suitable for tissue engineering and regenerative medical applications. This assay revealed a cell viability of ~ 75-80% for the HA-TIB-PBA and HA-TIB-Fru conjugates, similar to their non-labeled counterparts (**Figure S5**).

### Rheological properties, injectability and visibility with CT imaging of the iodine-labeled HA hydrogel

**Figure 3:**
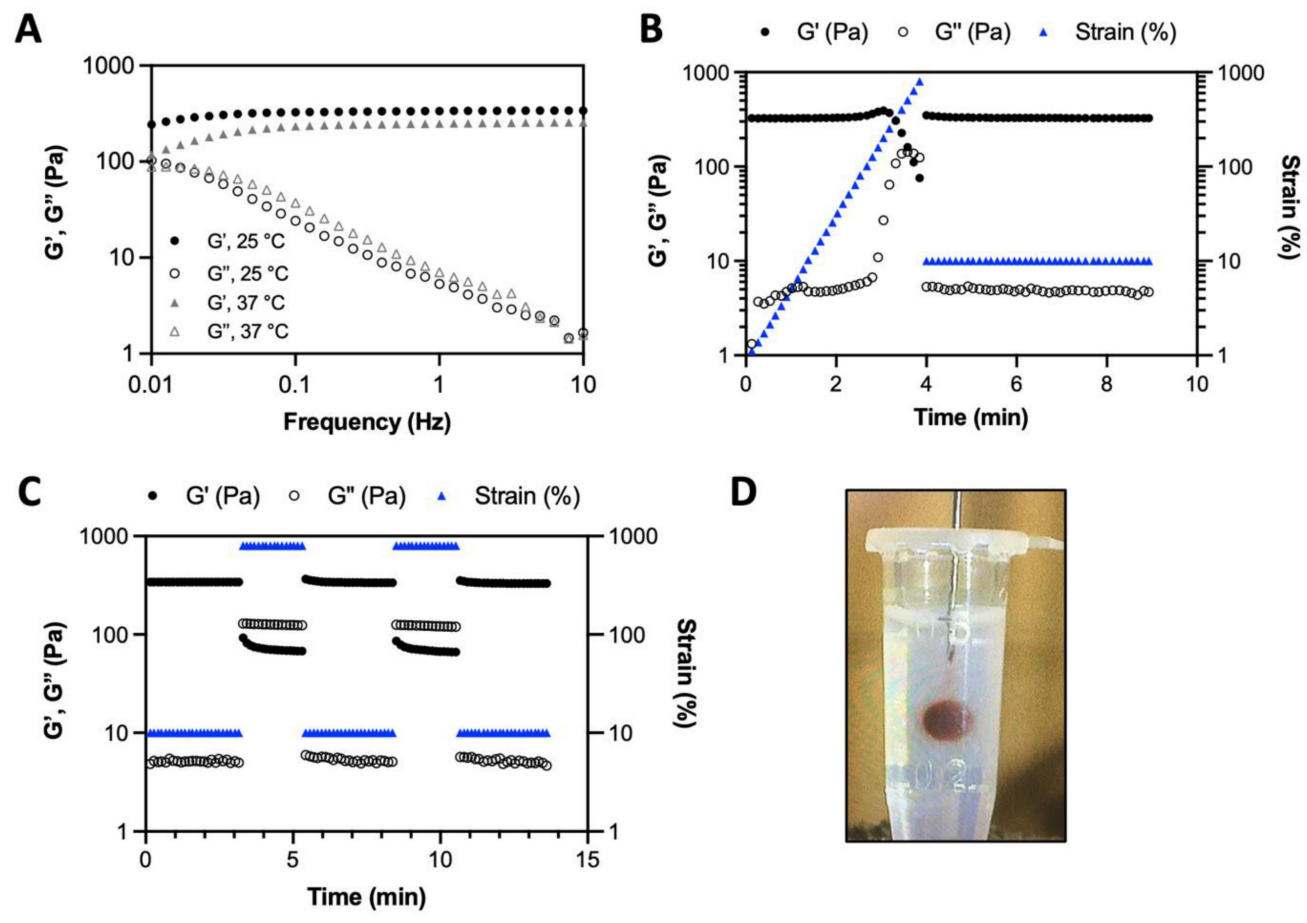
A) Frequency dependence of the storage modulus (G') and loss modulus (G'') of the HA-I hydrogel measured with 10% strain at 25° C and 37° C. B) Variation of G' and G'' when increasing strain values to 800% (hydrogel disruption), followed by reducing the strain to a constant value of 10% (linear viscoelastic region). C) Alternate step strain sweep tests with alternating strain deformations of 10 and 800% at a fixed frequency (1 Hz). D) Photo of hydrogel injection in an agarose phantom through a 26G (0.46 mm diameter) needle (neutral red was added to color the hydrogel for visualization only).

The iodine-labeled injectable HA (HA-I) hydrogel was produced by simply mixing thoroughly solutions of HA-TIB-Fru and HA-TIB-PBA in PBS (pH 7.4), at a total polymer concentration (*C_p_* = 18 g/L) and with a molar ratio of PBA-to-grafted fructose of 1. Benefiting from the rapid reaction kinetics of boronate ester formation 46, the gelation occurred immediately upon homogeneous mixing the two HA partners. Dynamic rheological analyses revealed a gel-like behavior (G' > G”) within the frequency window explored, as a result of formation of boronate ester crosslinks between the two HA partners (**Figure 3A**). This behavior is similar to that of the non-labeled HA hydrogel prepared by mixing HA-Fru and HA-PBA (C*_p_* = 12 g/L, **Figure S6**). As shown in **Figure 3B**, strain-dependent oscillatory measurements displayed a broad linear viscoelastic region with network failure at high strain (800%). This feature can be recognized as a benefit for the use of this hydrogel as synovial fluid supplementation in joints subjected to high-strain activities. In addition, the network was shown to immediately recover its rheological properties when the strain was reduced to 10%. Next, the gel was subjected to a series of two cycles of breaking and reforming, which consisted in applying large strain deformations (800%), intercalated with low strain deformations (10%) (**Figure 3C**). These strain-recovery experiments revealed full recovery of the gel network, demonstrating its self-healing property. Although dynamic rheological moduli and self-healing capacity are important parameters for determining injectability, injection tests of the HA-I hydrogel in an agarose-based tissue-mimicking phantom 47, 48 were also carried out to verify the suitability of the HA-I scaffold for intra-articular injection. To this end, the hydrogel (10 μL) was injected using a Hamilton syringe with a 26G needle, at a rate of 5 µL/min. As illustrated in **Figure 3D** and in the video (**Video S1**), the HA-I hydrogel stained in red (neutral red) could be injected with precision in the agarose phantom. Next, we examined the ability to visualize the HA-I hydrogel using synchrotron K-edge subtraction CT 49. KES imaging was first proposed by B. Jacobson in 1953 50. It uses two images acquired at different average energies, slightly below and slightly above the K-edge of the high Z-element of the contrast agent. Subtracting these images produces an image of the element of interest (here the contrast agent), while other anatomical or bony structures are eliminated because their attenuations remain almost constant 51. SKES-CT is the gold standard for this method, as the synchrotron allows monochromatic beams to be used, providing very high measurement accuracy. Furthermore, the high dose rate available at the synchrotron makes it possible to obtain high resolution quantitative images with high sensitivity, by increasing the radiation dose while maintaining reasonable acquisition times for preclinical studies. Unfortunately, this is at the expense of the dose received by the animal. The principle of imaging with SKES-CT that allows to distinguish several different materials in the field of view simultaneously is illustrated in **Figure S7**. As shown in this figure, the iodine map specifically depicted the HA-I hydrogel contrary to the agarose matrix which did not produce any signal in the iodine map, as expected. SKES-CT and the iodine labeling were used in the following parts to track directly the hydrogel within the joints without compromising the visualization of bone tissue.

### Preclinical studies

The next step consisted in imaging the radiopaque HA-I hydrogel *in vivo* after administration in the knee of mice. Experiments at the synchrotron were organized in three sessions: in the first one, knee samples of healthy mice were imaged *ex vivo* to ascertain the feasibility of the imaging approach (in line with the 3R principles of minimizing animal use). The second session was dedicated to *in vivo* imaging of a mouse model of OA in the first 72 h post-administration. The third session aimed at assessing i) the added value of imaging for the monitoring of hydrogel delivery and the long-term fate of the HA-I hydrogel, and ii) the therapeutic effects of the HA-I in a mouse model of OA in a 5-week follow-up study.

#### *Ex vivo* SKES-CT imaging in healthy knee joints

**Figure 4:**
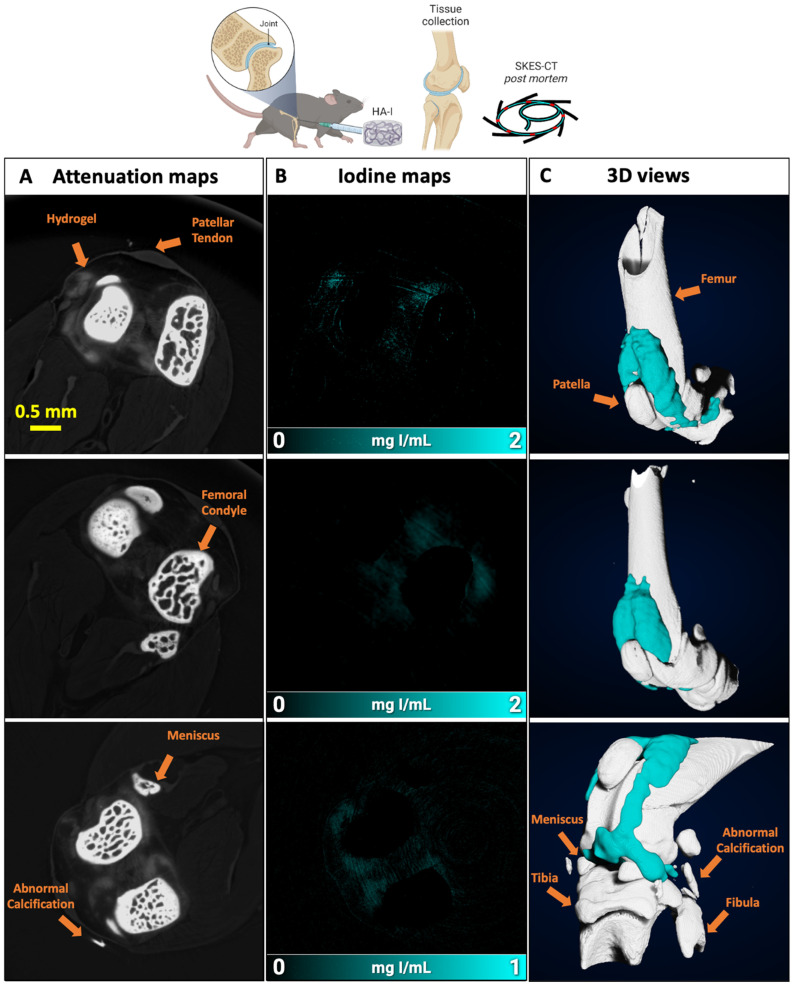
Imaging of the HA-I hydrogel in the knees of healthy mice with SKES-CT. Results for each knee are displayed on each row. A) Attenuation images (representative single slice from 3D data set). B) Corresponding iodine concentration maps. C) 3D view of segmented bone (white) and iodine (blue).

In the first session, 2.5 µL of HA-I hydrogel was injected into both knee joints of two healthy mice. The mice were sacrificed immediately after administration and SKES-CT imaging was performed *ex vivo*. Images showed that the HA-I hydrogel distributed around the patella (kneecap) as expected, demonstrating that the HA-I hydrogel can be used to monitor intra-articular delivery to the target site with CT (**Figure 4**). Iodine signal was present inside 3 out of 4 knee joints. This indicates a success rate of 75% for intra-articular injection of the HA-I hydrogel. This hypothesis is plausible given the difficulty associated with the intra-articular injection of small hydrogel volumes in mouse joints, and the success rate reported for conventional knee injections in humans with OA (71-93%) 52. The HA-I hydrogel volume calculated from the reconstructed 3D images were 1.3, 2.1 and 4.7 μL (mean ± standard deviation: 2.7 ± 1.4 µL). Considering the residual volume in the syringe and possible dilution of the hydrogel in the synovial fluid (synovial volume of 4-5 μL) 53, the volumes obtained from the SKES-CT images are in reasonable agreement with the actual injection volume (2.5 μL). These images suggest that the HA-I hydrogel form a stable gel structure after injection into the joint cavity, which is in line with the *in vitro* injection test carried out in the agarose hydrogel phantom (video S1). It should be noted, however, that the HA-I hydrogel is more susceptible to dilution in the SF than in the agarose gel due to its viscoelastic properties 54 contrary to the elastic behavior of the agarose gel 55. Such *in situ* volumetric detection would allow noninvasive monitoring of the HA-I hydrogel.

#### *In vivo* SKES-CT imaging of OA mouse knees in the first 72 h after injection

**Figure 5:**
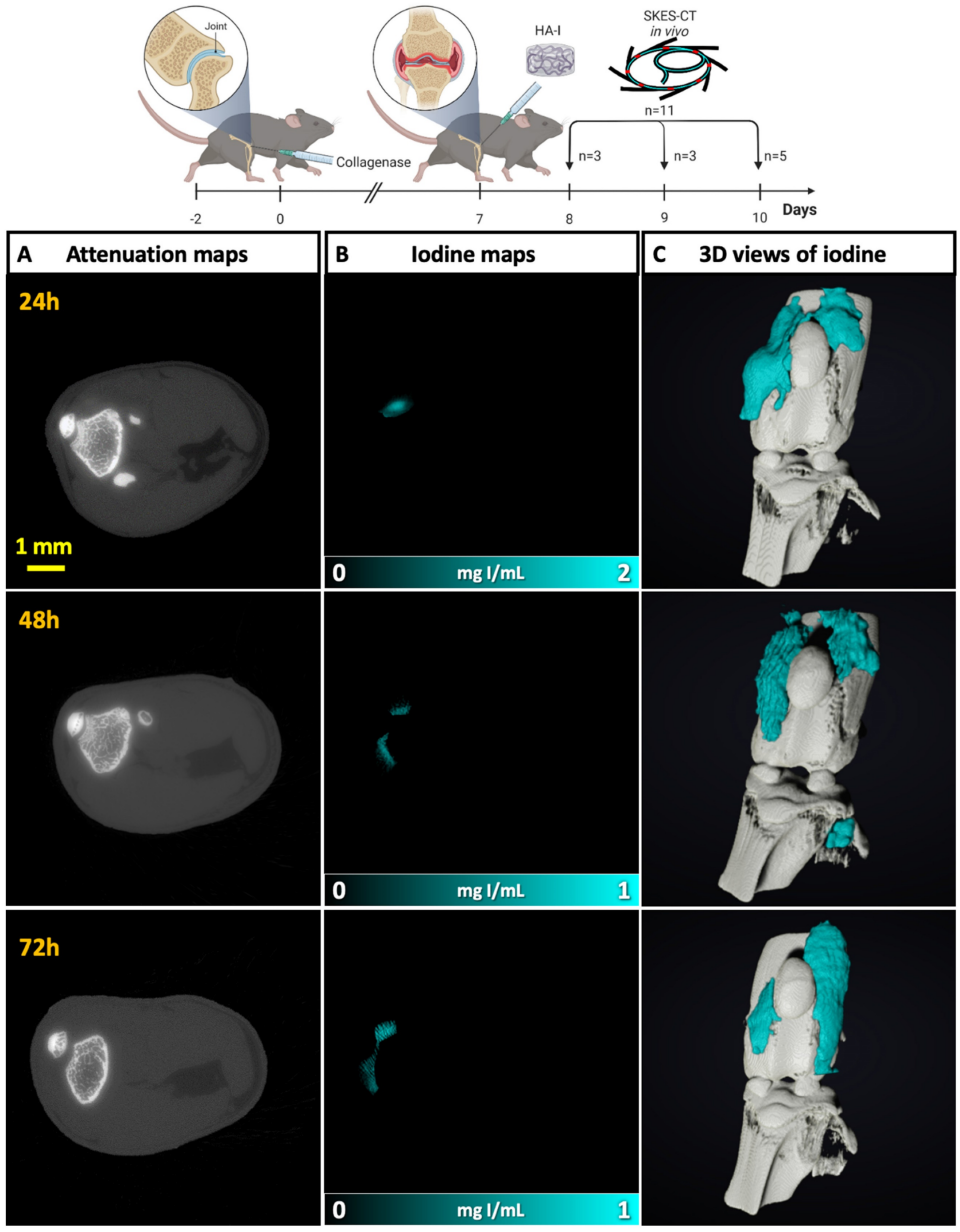
Imaging of the HA-I hydrogels with SKES-CT in the knees of OA mice. Results of 3 representative knees imaged at 3 different times post-administration are displayed on each row (24 h, n = 3; 48 h, n = 3; 72 h, n = 5). A) Attenuation images (representative single slice from 3D dataset). B) Corresponding iodine concentration maps. C) 3D view of segmented bone (white) and iodine (blue).

In the next session, we aimed to evaluate our imaging approach in the collagenase-induced OA (CIOA) model, which is described as the reference model of inflammatory OA 56, 57. The HA-I hydrogel was injected into the knees of OA mice (n = 11) and the 11 mice were imaged on different days post-injection to assess its distribution. More specifically, 3 mices were imaged at 24 h post-administration, 3 mices at 48 h and 5 mices at 72 h. As in the previous session, the HA-I hydrogel was found around the patella, suggesting a good precision of injection (**Figure 5**). The iodine signal was present in all knee joints at 24 h (3/3), in 2/3 knee joints at 48 h, and in 3/5 knee joints at 72 h. There are two possible reasons for the absence of iodine detection in some knees at 48 h and 72 h. The first one is failed intra-articular injection. Since the presence of iodine is observed in 8 out of 11 mouse knees, this would mean an accuracy rate of 73% for intra-articular injection of the HA-I hydrogel, consistent with the previous session. The second reason may be the elimination of hydrogel due to HA degradation. It should be noted, however, that the calculated hydrogel volumes in the mouse knees were in the same range for all time points (mean ± standard deviation for successful injections: 1.9 ± 1.3 μL at 24 h, 1.7 ± 0.1 µL at 48 h, and 1.7 ± 1.3 µL at 72 h, **Figure S8**). Although it is difficult to conclude because of the small number of animals, this trend invalidates the second explanation and suggests the stability of HA-I hydrogel during the first three days post-injection.

Last, the knees of these mice were sampled and imaged post-mortem with X-ray phase contrast tomography (XPCT), in order to obtain a ground truth 3D phase contrast image of the knee joints at the spatial resolution of 6 µm. The hydrogel distribution was readily visualized within the joint (**Figure S9** and **Video S2**) and was consistent with SKES-CT findings.

#### Evaluation of *in vivo* location/retention of the HA-I hydrogel following intra-articular injection and its therapeutic effect *in vivo* in the collagenase-induced OA model

**Figure 6:**
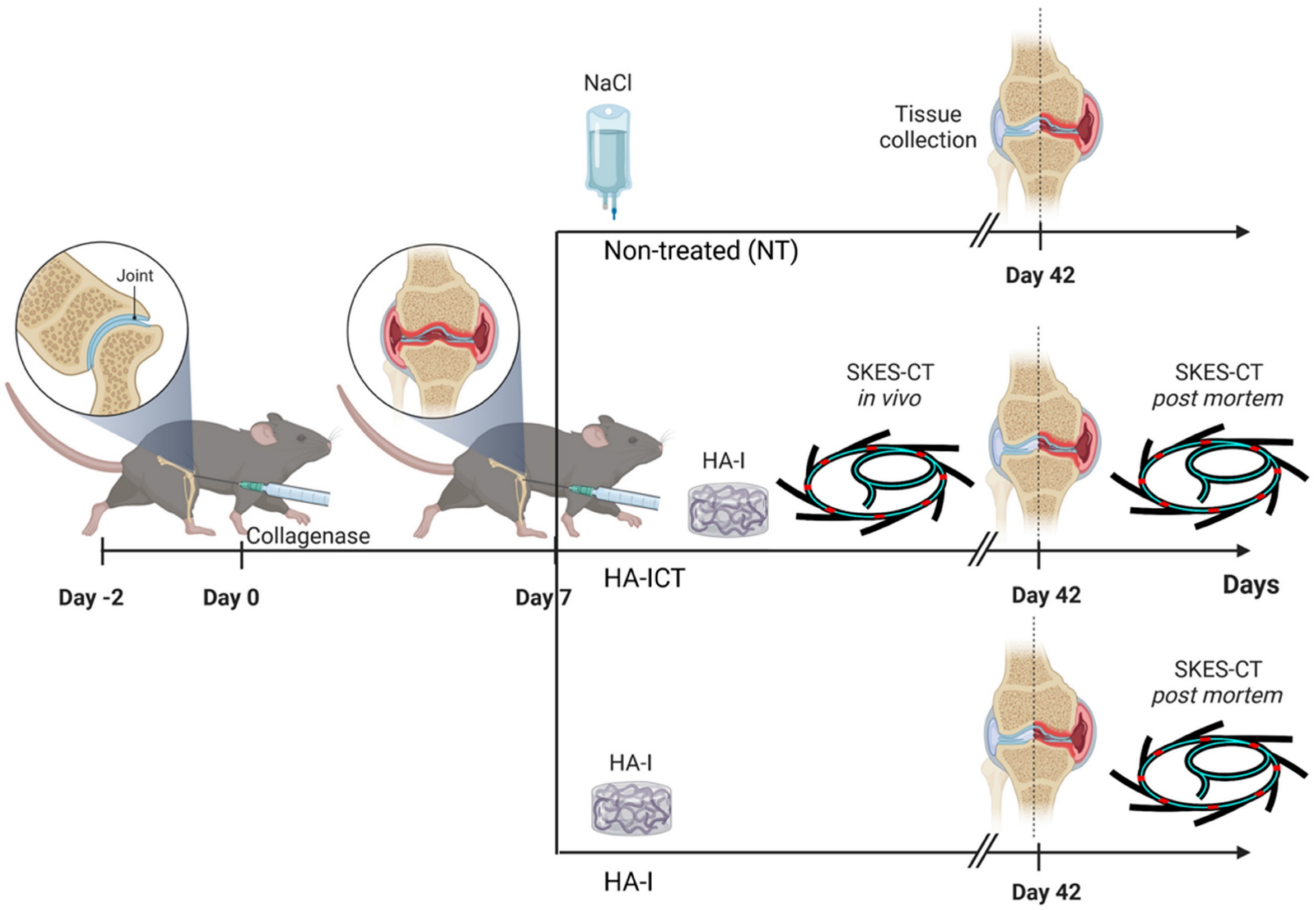
Animal experiment procedure to investigate the intra-articular location/retention of the hydrogel after injection and its therapeutic effect.

Finally, to investigate the intra-articular location/retention of the hydrogel after injection and its therapeutic effect, we combined SKES-CT imaging of the HA-I hydrogel with biological analyses of cartilage and bone degradation. As illustrated in **Figure 6**, collagenase-treated mice were divided into 3 groups: in non-treated (NT) group, mice received 2.5 μL saline by intra-articular route in the knee joint at day 7 (D7) following collagenase administration (n = 15); in HA-ICT group, mice received a single intra-articular injection of HA-I hydrogel (2.5 μL) in the knee joint at day 7 and, were imaged on both day 7 for delivery monitoring (immediately after administration) and day 42 post-mortem (n = 16); in HA-I group, mice received a single intra-articular injection of HA-I hydrogel (2.5 μL) in the knee joint at day 7 following collagenase administration and knees were imaged post-mortem at day 42 (n = 16).

**Figure 7:**
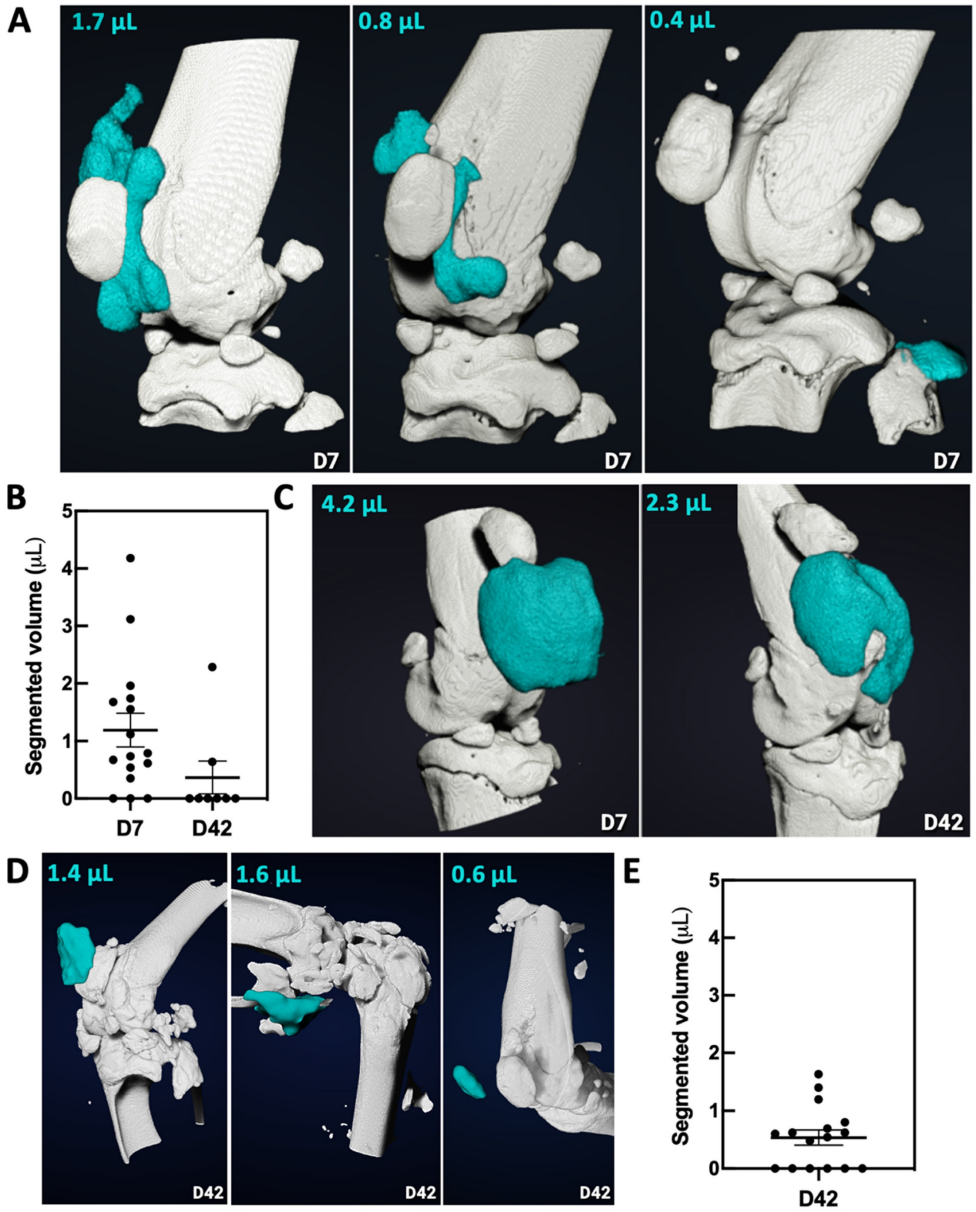
Imaging and quantification of the HA-I hydrogel with SKES-CT in the knees of OA mice (white for bone and blue for iodine). A) Three representative knees imaged on the day of injection (day 7, group HA-ICT). B) Quantification of the volume of HA-I hydrogel in knee joints at day 7 and day 42 in group HA-ICT (mean ± SEM). C) Images of the knee joint of a mouse taken at day 7 and day 42 (group HA-ICT). D) Three representative knees imaged at day 42 (group HA-I). E) Quantification of the hydrogel volume in knee joints at day 42 in group HA-I (mean ± SEM).

For the group HA-ICT, SKES-CT imaging on day 7 revealed that the HA-I hydrogel was present in 13 out of 16 knee joints on the day of injection (success rate of 81%), consistent with previous findings. Images showed again that the HA-I hydrogel distributed around the patella in 9/13 cases (**Figure 7A**). The HA-I hydrogel volume obtained from the SKES-CT images ranged from 0.4 to 4.2 μL with a mean of 1.5 μL (**Figure 7B**). Considering the residual volume in the syringe and possible gel dilution in the mouse knee joint, the values are fairly consistent with the actual volume of injection (2.5 μL). Post mortem imaging on day 42 was carried out on only 8 knees out of 16 due to technical issues. SKES-CT images revealed the presence of hydrogel in the knee joint of 2 mice with respectively 0.6 and 2.3 µL (mean ± standard deviation of 1.4 ± 1.1 µL for successful injections). **Figure 7C** shows the longitudinal follow-up of the mouse that still had 2.3-µL HA-I hydrogel at day 42 post-administration.

Figure 7D displays representative 3D images of HA-I hydrogel at day 42 in the group HA-I. For this group, the hydrogel could be detected post-mortem in 10 out of 16 animals at day 42 (**Figure 7E**). The HA-I hydrogel volume obtained from the SKES-CT images ranged from 0.5 to 1.6 μL with a mean ± standard deviation of 0.9 ± 0.4 μL.

**Figure 8:**
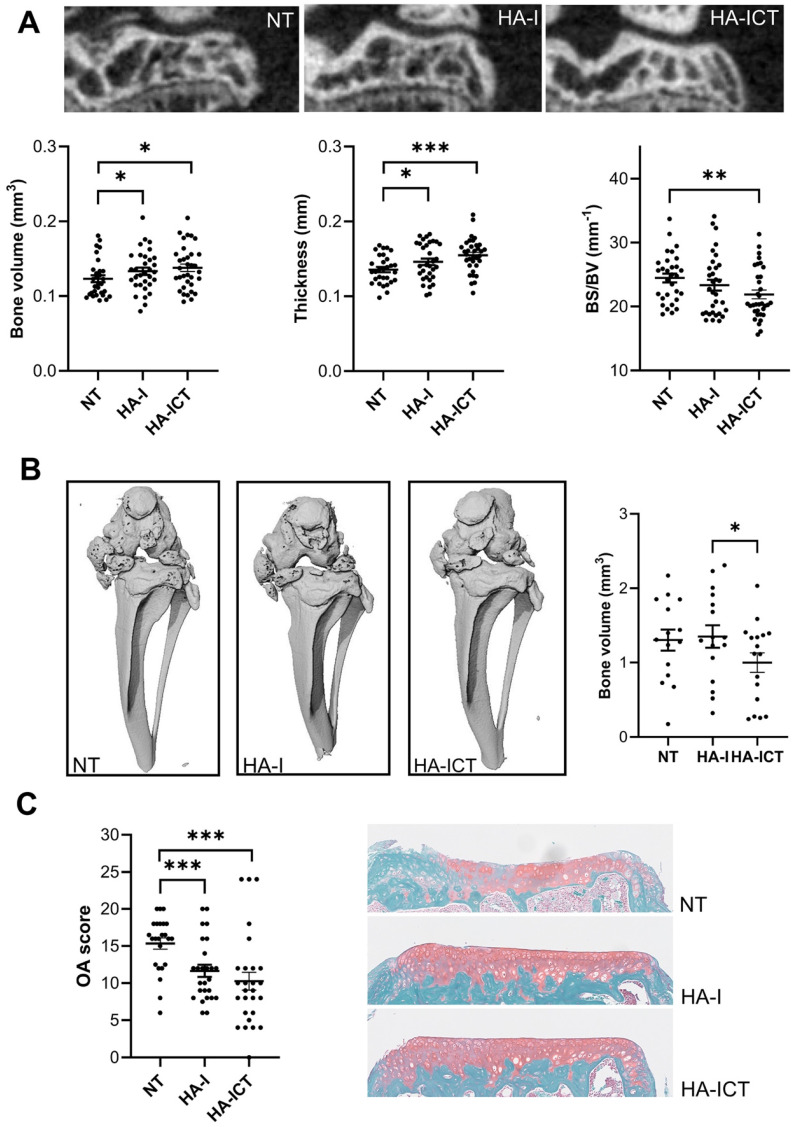
Protective effects of the HA-I hydrogel in the collagenase-induced osteoarthritis murine model. A) Representative 2D images of the lateral epiphysis of mice imaged post mortem by conventional µCT at day 42 (upper panel). Groups correspond to non-treated mice (NT) or mice injected with the iodinated HA gel with an additional SKES-CT *in vivo* imaging at day 7 (HA-ICT) or without (HA-I). Histomorphometric parameters of sub-chondral bone plates (lower panel; bone surface (BS), bone volume (BV)). B) Representative post mortem 3D conventional µCT images of the joints at day 42 showing ectopic calcifications of menisci and ligaments of the joint and quantification of calcified bone volumes. C) Osteoarthritis (OA) score and representative images of histological sections from the three groups of mice. Results are expressed as mean ± SEM. * *p* < 0.05; ** *p* < 0.01; *** *p* < 0.001 (Statistical analysis used the Mann-Whitney test (A,C: n = 30 including lateral and median plateaux; B: n = 15 entire joints).

In parallel, the effect of the hydrogel has been investigated on OA symptoms. At the bone level, several histomorphometric parameters differed between groups. The bone volume and thickness of sub-chondral plateaux were significantly higher in the HA-I and HA-ICT groups compared to the NT group while the surface degradation, evaluated by the bone surface/bone volume ratio, was significantly lower (**Figure 8A**). The calcification of menisci and ligaments in the peri-articular space, which is observed in the NT OA group, was significantly lower in the HA-ICT group (**Figure 8B**). Finally, the effect on articular cartilage was evaluated by histology. The degradation of cartilage surface was significantly lower in the two groups of HA-I and HA-ICT as shown by the representative images and OA score quantification (**Figure 8C**). Altogether, improvement of both bone and cartilage parameters was demonstrated with a trend to better results for the HA-ICT group.

To evaluate the added value of imaging for predicting therapeutic outcome, we correlated imaging and histological data. There was no correlation between the volume of hydrogel detected on imaging immediately after administration and the histological score of medial tibial plateau cartilage at day 42 (**Figure S10A**). In contrast, there was a negative association between the volume of hydrogel detected on imaging at day 42 and the histological score of medial tibial plateau cartilage at day 42 (**Figure S10B**). This suggests that the hydrogel tended to disappear faster in mice with more severe OA, regardless of the amount of hydrogel actually delivered, probably due to a pro-inflammatory environment sustained in time. To go a step further, we tested the hypothesis that individuals in which the hydrogel had totally disappeared at day 42 had a more pejorative outcome than individuals in which the hydrogel was still present at day 42. Indeed, mice in which the hydrogel was not seen at day 42 had the same histological score as non-treated animals while mice in which the hydrogel was still seen at day 42 had a significantly lower histological score, indicative of a better outcome (**Figure S10C**). Finally, we evaluated whether imaging was able to discriminate responders from non-responders to treatment. The histological score was 15 ± 4 in the non-treated group. Mice that had a histological score strictly superior to 11 (i.e. mean of non-treated group minus one standard deviation of non-treated group) were defined as non-responders to treatment. The volume of HA-I at administration was not statistically different between responders (1.5 ± 1.6 µL) and non-responders (1.7 ± 1.0 µL; *p* = 0.45) (**Figure S10D**). In contrast, the volume of HA-I that remained in the joint at day 42 was higher in responders (0.9 ± 0.8 µL) than in non-responders (0.3 ± 0.5 µL) (**Figure S10E**). This suggests that longitudinal imaging may provide a surrogate marker of response to treatment and thus, change patient management.

## Discussion

In this study, we designed and characterized a novel iodine-labeled injectable self-healing HA hydrogel for OA therapy. Our strategy relied on crosslinking HA with dynamic covalent (boronate ester) bonds that endow HA with unique mechanical features, such as viscoelastic properties and self-healing capability. The viscoelastic properties of SF are critical to its functions of lubrication and shock-absorption during walking and running 58. In OA, SF viscoelasticity and consequently, its ability to protect cartilage is dramatically lowered due to degradation of HA 59. Therefore, HA-based VS has been developed to restore these properties and relieve pain. Several studies showed that cross-linked HA formulations such as Hylan G-F 20 (Synvisc®, Genzyme Corp), are much more efficient in improving the rheological behaviour of OA SF than linear HA 59, 60. Moreover, in equine OA, highly viscoelastic HA formulations have been reported to provide longer lasting and greater levels of pain relief with fewer injections, when compared to HA products which were less viscoelastic 61. These results thus show significant advantages of crosslinked HA formulations as VS products in terms of performance and longevity compared to linear HA. However, covalent crosslinking of HA has some limitations in terms of injectability. In the case of the most extensively studied product Hylan G-F 20, for instance, crosslinked HA chains (Hylan B), which form an insoluble gel, are mixed with soluble high molar mass HA (Hylan A) to overcome this issue. On the other hand, only the soluble portion (Hylan A, representing 80% by volume of the product) has been shown to be functional with respect to CD44 receptor interaction 62. Yet, HA-CD44 binding has been shown to have numerous downstream effects that combat the symptoms of knee OA 63. The HA-I hydrogel developed in this work may be a promising alternative for VS as it combines highly viscoelastic properties with injectability thanks to its self-healing ability. The latter not only allows fast recovery of the hydrogel properties after injection, thereby ensuring local hydrogel confinement, but also enables cell migration and molecular diffusion 64. To our knowledge, there is only one example in the literature of the use of a self-healing hydrogel composed entirely of HA for OA treatment 43. This hydrogel, crosslinked by cooperative hydrogen bonding, was used at a HA concentration of 100 g/L which is much higher than that of the HA-I hydrogel (18 g/L) and Hylan G-F 20 (8 ± 2 g/L) 59. While both HA-I hydrogel and Hylan G-F 20 exhibit elastic behaviour (G' > G'') over a wide range of frequency, the G' modulus at 2.5 Hz (value of G' in the plateau region) of the HA-I formulation is ~ 3 times higher than that of Hylan G-F 20 at 25° C. As the plateau modulus scales with the number density of elastically active chains, the higher G' value of the HA-I hydrogel may be related to both higher crosslink density and HA concentration. Noteworthy is the fact that iodine labeling did not alter the self-healing and injectability properties of the HA hydrogel. In addition, we verified *in vitro* that labeling of the HA gel precursors with the clinical iodine-based contrast agent (AcTIB) did not impact viability of adipose-derived stromal cells. More importantly, our study provides proof-of-concept that iodine labeling allowed to monitor the hydrogel delivery and retention *in vivo* in mouse knees up to 5 weeks post-administration. Taken together, our data indicate that the HA-I hydrogel we developed presents stable iodine labeling as well as excellent properties for intra-articular injection with good precision as demonstrated by SKES-CT imaging. To the best of our knowledge, this represents a technological first in the field of HA-based VS. Analysis of more than twenty publications on landmark-guided knee injections of VS products revealed varying accuracy depending on approach and experience of injector, with the superolateral patellar approach in the extended knee being the most accurate in patients (87% accuracy) 65, 66. These data underscore the need to standardize the procedure to ensure patient comfort and safety, and to achieve effective pain relief. Ultrasound-guided injection has been recommended to ensure precise needle placement, improving the success rate and also preventing complications associated with the procedure 67, 68. However, other imaging modalities such as fluoroscopy, which requires an iodinated contrast medium to highlight the joint cavity before administering HA, must be used to verify injectate distribution patterns 69. Although such an approach is valuable for monitoring the delivery of HA in the joint space 69, it does not allow long-term visualization of the hydrogel. In the present study, delivery monitoring of the radiopaque hydrogel using SKES-CT revealed that it was precisely injected into joints of OA mice (hydrogel visualized in 13 of 16 mice, i.e. 87%). This value was similar to that mentioned above for humans, despite the difficulty associated with the intra-articular injection of small hydrogel volumes in mouse joints. Moreover, the volume of the hydrogel calculated on the basis of 3D reconstruction provided valuable information about the quantity of hydrogel actually reaching the knee joint, which might also prove useful for treatment standardization 70.

One of the major issues in the field of VS is to determine the fate of the hydrogel on the long-term. The long-lasting radiopacity of the HA-I hydrogel allows to address this issue. Our data indicated that the HA-I hydrogel was still present within the joint of mice for at least 5 weeks post intra-articular injection. The HA-I hydrogel compares favourably with the duration of ~ 4 weeks reported for Hylan G-F 20 in the healthy joint of rabbit 23. The Hylan B gel component of Hylan G-F 20 is the main contributor to this long residence time as its half-life (8.8 days) was found to be much longer than the half-life of Hylan A fluid (1.5 days) 23. This result suggests that dynamic covalent crosslinking is an attractive strategy to prolong the residence time of HA in the joint.

In addition to the exceptional longevity of the HA-I hydrogel coupled with its outstanding mechanical properties, its ability to slow the progression of cartilage and bone degeneration has been demonstrated. Indeed, the sub-chondral bone tissue was protected and the OA score indicated cartilage protection in the groups of mice that have received the HA-I hydrogel. This protective effect of HA-I hydrogel was expected since the role of HA in OA when used as single injections or in combination therapies has been widely discussed and its lubricating, anti-inflammatory and chondroprotective effects have made it an attractive option for the treatment of rheumatic diseases and notably OA 71-73. Here, we showed that the protective effect was even higher in the HA-ICT group, which was imaged *in vivo* on day 7 for monitoring delivery of the hydrogel. The better therapeutic outcome observed in the HA-ICT group may be related to the synergistic anti-inflammatory effect of the HA hydrogel and the X-ray dose delivered during CT acquisitions on day 7. Indeed, in these experiments, the X-ray dose for *in vivo* imaging was relatively high (~ 2.4 Gy). This is due to several factors: the high resolution (22 microns), the low detector efficiency (30%) 74 and the high signal-to-noise ratio needed to detect small concentrations of iodine (down to 0.2 mg/mL). The radiation dose could have been reduced by 30% if shutter had been used to protect the animal during the reading time of the camera (not available at the time of the experiment). Our aim at term is to use spectral (dual-energy or photon counting CT) to monitor the hydrogel in larger animal models so that the X-ray dose will not interfere with hydrogel treatment. In the present study, we used SKES-CT to provide a proof-of-concept of the value of imaging for monitoring the delivery of the HA-I hydrogel in the mouse model of OA. In the HA-ICT group, the dose delivered during CT imaging is close to a radiotherapy dose fraction.

It has been reported that low dose radiation therapy has strong anti-inflammatory effects and OA of large and small joints has been shown to benefit from radiation therapy in patients 75. Several mechanisms have been described, including macrophage polarization toward an anti-inflammatory phenotype, production of anti-inflammatory cytokines, reduced production of reactive oxygen species (ROS) and increased apoptosis of pro-inflammatory cells. In animal models, low doses of 0.5 to 1.5 Gy and total doses of 2.5 to 7.5 Gy were histologically shown to have an anti-inflammatory effect, especially in inflammatory arthritis models 75, 76. It should be noted that the first SKES-CT imaging at day 7 may also have impacted the hydrogel degradability over the 42 days of follow-up. Indeed, the hydrogel was detected in 25% in the HA-ICT group, while it was found in 62% for the HA-I hydrogel. This difference may be attributed to activation of chondrocytes and synoviocytes by X-rays, promoting secretion of molecules that degrade HA 77. Further *in vitro* experiments should be designed to decipher the exact mechanisms leading to faster or slower degradation of the hydrogel *in vivo*.

Taken together, these results indicate that the anti-inflammatory effect of the HA hydrogel does not need to be present on the long term but in the first few days following administration to act during the inflammatory phase in this OA model. Since this phase lasts for at least 10 days following collagenase injection 56, the remanence and stability of the hydrogel for at least 72 h is an important factor contributing to its therapeutic effectiveness. Iodine labeling of the hydrogel is a precious tool to better understand and design VS therapy in OA. Nevertheless, further studies focusing on lubricative, adhesive, and stability attributes 60, 78 are needed to deepen our understanding of the mode of action of the HA-I hydrogel, thereby contributing to optimize the hydrogel formulation.

Our data further show that the quantification of iodine signal at day 42 by imaging can differentiate between responders and non-responders to HA-I hydrogel treatment. Mice treated with HA-I that have the same outcome as non-treated mice displayed a significant decrease in iodine signal compared to mice that had an improved outcome. The volume of hydrogel in both experimental groups was not significantly different at administration, excluding differences in iodine content as the underlying cause for the observed signal differences. Longitudinal imaging thus provides an early biomarker that can help stratify responders from non-responders in the first weeks post-VS. This has the potential to change patient management before the worsening of clinical symptoms, by repeating hydrogel administration with optimal injection intervals fine-tuned through longitudinal imaging.

To foster clinical translation, our results call for further research validation with larger animal model to test efficacy, safety and development of personalized treatment plans. Non-inferiority trials (i.e. trials comparing the novel hydrogel to the reference VS treatment) will inform us about the feasibility of replacing clinically-approved hydrogels for OA treatment. For safety, it is noteworthy that no adverse effects have been observed on the short-term or long-term in the 60 mice who received intra-articular injection of the HA-I hydrogel. In addition, the iodine contrast agent used to label the HA hydrogel precursors is derived from a molecule used in clinic. Severe allergic reactions to intra-articular contrast agent administration are rare enough to be case reportable, especially when compared to intra-vascular administration 79. Although thorough toxicity evaluation should be performed prior to clinical use, these findings indicate that this new radiopaque HA hydrogel is of well biocompatibility. Long-term studies will be needed to comprehend the chronic effects of the hydrogels and their degradation over time. Imaging with a spectral CT will also be an important step to confirm and extend our findings in the clinical setting. Dual energy and spectral photon counting CTs both generate iodine maps and they have increasing clinical availability. One of their advantages is the reduction of radiation exposure due to noise reduction, thus allowing repeated exams. There are also a few SPCCTs that are being developed to image small animals 80: it would be interesting to evaluate their performance in comparison with SKES-CT. Finally, another innovative application of the hydrogel in OA would be to use it to encapsulate stem cells 81. This will be the subject of a subsequent publication.

## Conclusion

This study demonstrates that this new radiopaque HA hydrogel crosslinked by dynamic covalent bonds offers great potential for the personalized treatment of knee osteoarthritis. Its outstanding features, i.e. long-lasting radiopacity and self-healing ability, combined to its ability to slow the progression of cartilage and bone degeneration, addresses the unmet need for a theranostic VS product to ensure patient comfort and safety, and to achieve effective pain relief. Our data demonstrated the promising beneficial effect of the HA-I hydrogel in a mouse model of OA. This theranostic tools provided novel insights into the mechanism of action of VS, showing that neither the volume of HA-I at delivery nor its long-term remanence were major determinants of treatment success. In turn, the rate of HA-I disappearance seemed to predict response to treatment, probably because a fast disappearance is an indirect measure of *in situ* inflammation. This theranostic hydrogel appears as a promising candidate for precision medicine in OA.

## Experimental section

### Materials

Hyaluronic acid sodium salt samples possessing a weight-average molar mass (M_w_) of 390 and 120 kg/mol (HA390 and HA120, respectively) were purchased from Contipro France. The molar mass distribution and the weight-average molar mass of these samples were determined by size exclusion chromatography using a Waters GPC Alliance chromatograph (USA) equipped with a differential refractometer and a light scattering detector (MALS) from Wyatt (USA); the solution was injected at a concentration of 1 mg/mL in 0.1 M NaNO_3_, at a flow rate of 0.5 mL/min and at a column temperature of 30° C. The dispersity (*Đ*) of the samples is M_w_/M_n_ ≈ 1.5-2. The overlap concentrations C* for HA390 and HA120 in PBS buffer at 25° C, are equal, to ~ 1.1 and ~ 2.9 g/L, respectively. This value was derived from the intrinsic viscosity 82 assuming that C*[η] is about unity 83. 1-Amino-1-deoxy-D-fructose hydrochloride (fructosamine) was supplied by Biosynth. 3-Aminophenylboronic acid hemisulfate salt (APBA), 4-(4,6-dimethoxy-1,3,5-triazin-2-yl)-4-methylmorpholinium chloride (DMTMM), phosphate-buffered saline (PBS), 3-acetamido-2,4,6-triodobenzoic acid bis(2-hydroxyethyl)-ammonium salt, *N*-Boc-ethylenediamine, 1-[bis(dimethylamino)methylene]-1H-1,2,3-triazolo(4,5-b)pyridinium 3-oxide hexafluorophosphate (HATU), agarose (Reference A9539), and other chemicals were purchased from Sigma-Aldrich and were used without further purification. Therapeutic grade human adipose-derived stromal cells were provided from EFS (“Etablissement Français du Sang”) for *in vitro* experiments. Platelet lysate and heparin 5000 U/mL, beta fibroblast growth factor (βFGF), 3-(4,5-dimethylthiazol-2-yl)-2,5-diphenyl tetrazolium bromide (MTT), Dulbecco's phosphate buffer saline, and ⍺-MEM (⍺-Minimum Essential Media) were purchased from ThermoFisher Life Science. *N*-(2-aminoethyl)-3-acetamido-2,4,6-triiodobenzamide (AcTIB-NH_2_) was synthesized as described in Supporting Information (**Figure S1**). Non-labeled HA-PBA (DS_PBA_ = 0.15) and HA-Fru (DS_Fru_ = 0.15) were prepared from HA390 as described previously 45.

### Synthesis of the Iodine-labeled HA gel precursors

Firstly, HA-TIB derivatives **3** with a molar mass of 390 and 120 kg/mol were synthesized by an amide coupling reaction between *N*-(2-aminoethyl)-3-acetamido-2,4,6-triiodobenzamide (AcTIB-NH_2_, **2**) (0.177 g, 0.30 mmol) and, respectively, HA390 and HA120 (0.200 g, 0.50 mmol) in a water/DMF (3/2, v/v) mixture containing DMTMM (0.10 g, 0.36 mmol). The reaction was conducted at pH 6.5 for 48 h at room temperature. After purification by ultrafiltration using deionized water, the iodine-labeled HA390 and HA120-TIB derivatives **3** were recovered by freeze-drying with 84 and 80% yields, respectively. The DS of HA390-TIB and HA120-TIB were found to be, respectively, 0.26 and 0.20 from ^1^H NMR analyses. In a second step, the derivatives were reacted with fructosamine and APBA according to the following conditions. For the synthesis of HA-TIB-Fru, fructosamine (0.012 g, 0.05 mmol) was added to a water/DMF (3/2, v/v) mixture containing DMTMM (0.090 g, 0.32 mmol) and HA390-TIB (0.18 g, 0.32 mmol), and the pH was adjusted to 6.5. For the synthesis of HA-TIB-PBA, APBA (0.007 g, 0.036 mmol) was added to a water/DMF (3/2, v/v) mixture containing DMTMM (0.100 g, 0.36 mmol) and HA120-TIB (0.16 g, 0.36 mmol) and the pH was adjusted to 6.5. After stirring for 24 h at room temperature, both HA derivatives were purified by ultrafiltration (membrane MWCO 10 kDa) using deionized water and were recovered by freeze-drying with 90% yield. The DS_Fru_ of the HA-TIB-Fru derivative **5** was found to be 0.15 and the DS_PBA_ of the HA-TIB-PBA derivative **7** was found to be 0.10 from ^1^H NMR analyses.

### Preparation of the HA-I and HA-ref hydrogels for rheometry

The HA-I hydrogel was prepared by mixing solutions of HA-TIB-PBA **7** and HA-TIB-Fru **5** in PBS (pH 7.4) at a total polymer concentration of 18 g/L and with a boronic acid/sugar molar ratio of 1/1, using a double-barrel syringe equipped with an extruder (MEDMIX, Switzerland). The concentration of 18 g/L was determined based on conditions previously used to prepare a non-labeled HA-PBA/HA-Fructose hydrogel (storage modulus G'_1Hz_ ~425 Pa at 25° C) from HA derivatives with a HA molar mass (M_w_) of 360 kg/mol 45. The latter was typically prepared at a total polymer concentration (*C_p_* ) of 15 g/L, which is ~12.5-fold the overlap concentration of initial HA360 (C* ~1.2 g/L). Since both HA hydrogel precursors have the same molar mass, the initial concentration of each compound was approximately 15 g/L. In the present study, the HA-I hydrogel was prepared from HA derivatives with HA molar masses of 390 kg/mol and 120 kg/mol. Since the HA sample used to prepare the HA-TIB-Fru derivative had a molar mass (M_w_ = 390 kg/mol) close to that in previous published work, it was used at a concentration of 15 g/L to prepare the hydrogel. Regarding the HA-TIB-PBA (prepared from HA120), it was used at a concentration of 21 g/L to prepare the hydrogel, which is ~7.2-fold the C* value of initial HA120. This compound was used at this concentration in order to obtain a dynamic storage modulus (*G'*) of the same order of magnitude of the HA-PBA/HA-Fructose hydrogel published previously 45. The hydrogel was directly transferred to the plate of the rheometer. The HA-ref hydrogel was prepared by mixing solutions of HA-PBA and HA-Fru in PBS (pH 7.4) at a total polymer concentration of 12 g/L. These HA derivatives, which were synthesized from HA390 (C* ~1.1 g/L), were solubilized at this concentration to obtain a dynamic storage modulus (*G'*) of 425 Pa.

### Agarose gel preparation and injection tests

Agarose gels were prepared by solubilizing agarose (300 mg) in 50 mL of PBS (pH 7.4) under stirring at 95° C for 10 min. The agarose solution was then poured in an Eppendorf^®^ tube and the sample was kept at 4° C for 24 h before the injection tests. The latter were carried out with a TJ-1A syringe pump controller (Aniphy, USA), at a rate of 5 µL/min).

### NMR spectroscopy

^1^H NMR spectra were recorded at 25° C or 80° C using a Bruker AVANCE III HD spectrometer operating at 400.13 MHz (^1^H). ^1^H NMR spectra were recorded by applying a 90° tip angle for the excitation pulse, and a 10 s recycle delay for accurate integration of the proton signals. Deuterium oxide (D_2_O) and deuterated dimethylsulfoxide (DMSO-d6) were obtained from Euriso-top (Saint-Aubin, France). Chemical shifts (δ in ppm) are given relative to external tetramethylsilane (TMS = 0 ppm) and calibration was performed using the signal of the residual protons of the solvent as a secondary reference. All NMR spectra were analyzed with Topspin 4.3.0 software from Bruker.

### Rheological analysis

Dynamic rheological experiments were performed using a strain-controlled rheometer (ARES-RFS from TA Instruments) equipped with two parallel plates. All the dynamic rheological data were checked as a function of strain amplitude to ensure that the measurements were performed in the linear viscoelastic region. The parallel plate on which samples were placed has a diameter of 25 mm. The distance between the plates was 0.25 mm. A thin layer of low-viscosity silicone oil (50 mPa s) was applied on the exposed surface of the samples, to prevent water evaporation. The details of the rheological measurements were as follows: 1) oscillatory frequency sweep (0.01-10 Hz) experiments were performed within the linear viscoelastic range (strain fixed at 10%) to determine the frequency dependence of the storage (G') and loss (G”) moduli; 2) oscillatory amplitude sweep experiments at 1 Hz were carried out to determine the linear-viscoelastic range of the hydrogel networks and the yield stress. They were immediately followed by time sweep experiments at 1 Hz and a strain of 10% (linear viscoelastic region) to monitor the recovery of the rheological moduli; 3) alternate step strain sweep tests consisted in applying alternating strain deformations of 10 and 800% with a duration of 3 and 2 min, respectively, at a fixed frequency (1 Hz).

### * In vitro* cytotoxicity assay

Cytotoxicity studies were performed by a MTT assay with hASCs following conditions described previously 84. Human ASCs used in this study were isolated from human fat tissues after surgeries, then purified against any diseases and viruses. All experiments were performed using hASCs at passage P2-P3. Cells were cultured onto T175 flasks to reach 90-95% confluency in a ⍺-MEM supplemented with 3% platelet lysate and 1% heparin 5000 U/mL without antibiotics (penicillin/streptomycin). Cells were then trypsinized, pelleted and re-suspended into a growth media for cell counting. 2 × 10^3^ hASCs were incubated in 96-well plates with individual solutions of HA derivatives (HA-PBA, HA-Fru, HA-TIB-PBA, HA-TIB-Fru) and native HA in standard growth media. Cells were also incubated with solutions of the iodine contrast agent AcTIB at different concentrations in a 10% dimethylsufoxide (DMSO) + cell growth medium (from [I] = 1.30 mg/mL to [I] = 6.30 mg/mL) to assess the iodine-dose effect. After incubation at 37° C for 72 h, a MTT solution was added in each well at a final concentration of 0.5 g/L. After 2 h, the incubation media was removed and the blue MTT-formazan product was extracted with DMSO. After 15 min extraction at room temperature, the absorbance of the formazan solution was measured at 570 nm. The percentage of living cells was calculated based on values of absorbance measured for cells cultured only in growth media. The experiment was repeated 3 times independently.

### Animal experiments

All experimental procedures involving animals and their care were carried out in accordance with the European regulations for animal use (EEC Council Directive 2010/63/EU). An acclimation period of at least 7 days was observed before the start of the study. For evaluating the therapeutic effects of the hydrogel, a priori sample size was determined as 15 mice per group based on previous studies showing a therapeutic effect in this model 85. Data analyses were performed blindly. Schematics depicting the experimental procedures were created in Biorender.com.

The study was approved by the French ministry of research after evaluation by local ethical committees (APAFIS agreement ##35861-2022031115332865 and #7457-2016110414498389) where the CIOA model was performed and treatments were administered, and #31781-20210520132410 where knees were imaged with SKES-CT *ex vivo* and *in vivo.* C57BL/6J mice (age at reception: 10 weeks, body weight: 20-25 g) were purchased from Charles River Laboratory (L'Arbresle, France). The animals were housed in a temperature- and humidity-controlled environment (21 ± 3° C), with a 12 h light-dark cycle, free access to food and water and nest material according to the involved animal welfare units. A total of 60 mice was used in the study.

CIOA was induced as previously described 85. In brief, right knee joints of mice were injected with 1 U type VII collagenase from *Clostridium histolyticum* (Sigma-Aldrich) in 5 μL of saline via a 25G (0.51 mm diameter) needle at day 0 and day 2, causing alteration of the ligaments and local instability of the joint. All surgery was performed under isoflurane gas anesthesia, and all efforts were made to minimize suffering. For the first experiment (*ex vivo* imaging), healthy mice (n = 2) received intraarticular injections of 2.5 µL of HA-I hydrogel. Mice were sacrificed immediately post-injection and the joints were collected, fixed in formaldehyde solution (3.7%) then embedded in an 1% agarose gel for *ex vivo* imaging. For the second experiment (short term follow-up), a group of 11 mice with OA received the 2.5 μL of HA-I hydrogel at day 7 post-induction. SKES-CT imaging was performed on lived animals in the first 72 h following administration (24 h: n = 2, 48 h: n = 4 and 72 h: n = 5). For the third experiment (long-term follow-up), CIOA mice were randomized into 3 groups: (1) mice received 5 μL saline by intra-articular route in the right knee joint at day 7 (NT group, 15 mice) and were sacrificed at day 42; (2) mice received a single IA injection of HA-I hydrogel (2.5 μL) in the right knee joint at day 7 and were imaged *in vivo* immediately after hydrogel administration. They were sacrificed at day 42, knees were prepared as described above and imaged post-mortem (group HA-ICT, 16 mice); (3) mice received a single IA injection of HA-I hydrogel (2.5 μL) in the right knee joint at day 7. They were sacrificed at day 42 and knees were imaged post-mortem at day 42 as in group 2 (group HA-I, 16 mice). All animals were included in the study (no exclusion criteria).

For *in vivo* imaging, mice were anesthetized by intraperitoneal injection of ketamine and xylazine (100 and 10 mg/kg respectively) secured on a home-made 3D printed bed with the right knee in extension. The bed was then disposed in the imaging chamber on the rotating device in front of the light beam. At the end of the imaging session, mice recovered under supervision in a warmed chamber after subcutaneous injection of 1 mL of saline.

### Imaging of mice with a conventional micro-CT

At euthanasia, paws were recovered and fixed in 4% formaldehyde. For bone analysis, hind paws were scanned in a Micro-Computed Tomography (µCT) scanner (SkyScan 1176, Bruker, Kontich, Belgium) and 3D image stacks were reconstructed using the NRecon software (Bruker). The quantification of the subchondral bone of the tibia and calcification of the meniscus and ligaments was performed using the CTAn software (Bruker). Reconstructed 3D images of joints were obtained using Avizo software (Avizo Lite 9.3.0, FEI, France).

### SKES-CT and material decomposition

The SKES-CT acquisitions were performed on the biomedical beamline ID17 of the European synchrotron radiation facility (ESRF). The gap of the wiggler (B_max_ of 1.4T) was set at 80 mm. The beam was filtered by 0.8 mm of vitrous carbon, 2.5 mm aluminium and 3 cm plexiglass. A double bent Laue monochromator was used to produce monochromatic X-ray beams (∆E/E = 0.1%) that could be tuned below or above the K-edge of iodine (33.2 keV) at 32.2 keV and 34.2 keV. The distance between the X-ray source and the sample was 150 m and the sample to detector distance was 3.5 m and the beam height was 7 mm. The detector was a PCO Edge 5.5 camera coupled to a 60 µm thick Gd_2_O_2_S:Tb Scintillator (quantum efficiency of about 30% at 33 keV 74). The measured pixel size was 22.22 µm. The X-ray dose rate was measured using an ion chamber (UNIDOS PTW 31 002, Freiburg, Germany) and an unidos electrometer, and converted to dose in water. The dose rate in water was 0.1 Gy/s at 200 mA synchrotron ring current. The acquisitions were performed over 360 degrees using 1200 projections and an integration time of 10 ms per projection, resulting in a total dose in water of 2.4 Gy (2 images). The material decomposition process proposed by Granton et al. 86. was used to obtain the concentration maps, using images obtained above and below the K-edge of iodine, and the asumption that each voxel consists of only 3 materials: iodine, tissue or bone. The *ex vivo* knee samples were imaged with an isotropic resolution of 6.5 µm. Mice imaged *in vivo* reached an isotropic resolution of 13.3 µm.

### Segmentation method

The segmentation method is described in detail in a previous work 87. Briefly, a thresholding technique was used. The iodine threshold was set at 0.25 mg/mL. Morphological opening with structuring element of radius 2 pixels is performed. For knee segmentation, we performed a connected component analysis to keep only relevant objects. Three-dimensional reconstructions were generated with Dragonfly imaging software (https://www.theobjects.com/dragonfly/index.html). The volume of segmented iodine signal was used as the imaging endpoint.

### Histological analysis

After µCT analysis, hind paws were decalcified using 5% formic acid at room temperature for 2 weeks and then embedded in paraffin. Frontal sections of tibias were cut (3 slices of 7 µm each 100 µm; first section at 50 µm below the cartilage surface) and stained with safranin O and fast green. Cartilage degradation was quantified using the modified Pritzker OARSI score.

### Statistical analysis

Statistical analyses were performed using the GraphPad 9 Prism Software. Data distribution was assessed using the Shapiro-Wilk normality test and the Mann-Whitney test was used to compare the treated group to the NT control group. Data are presented as mean ± SEM. * *p* < 0.05; ** *p* < 0.01; *** *p* < 0.001.

## Supplementary Material

Supplementary figures.

Supplementary video 1.

Supplementary video 2.
