# Supplementary material for: A self-healing radiopaque hyaluronic acid hydrogel as a new injectable biomaterial for precision medicine in osteoarthritis
Source: Theranostics. 2025 Mar 10;15(9):4054–73. doi: 10.7150/thno.104551 (PMC11980665; doi:10.7150/thno.104551)
Supplement: Supplementary file 1 — Supplementary figures. [file thnov15p4054s1.pdf]

Supporting Information

A self-healing radiopaque hyaluronic acid hydrogel as a new injectable biomaterial for precision medicine in osteoarthritis

Moustoifa Said[1,2†], Clément Tavakoli[3,4†], Chloé Dumot[3,5], Karine Toupet[6], Cécile Olivier[4], Alexia Gilles[6], Marie Maumus,[6] Yuxi Clara Dong[7], Nora Collomb[2], Céline Auxenfans[8], Anaïck Moisan[9], Bertrand Favier[10], Benoit Chovelon[11,12], Emmanuel Luc Barbier[2], David Peter Cormode[7], Emmanuel Brun[4], Hélène Elleaume[4], Marlène Wiart[3], Olivier Detante[2,13], Claire Rome[2], Danièle Noël[6], Rachel Auzély-Velty[1]**

1. Univ. Grenoble Alpes, Centre de Recherches sur les Macromolécules Végétales (CERMAV-CNRS), 38041 Grenoble, France
2. Univ. Grenoble Alpes, Inserm, U1216, Grenoble Institut Neurosciences, 38000 Grenoble, France
3. Univ. Lyon 1, Inserm U1060, CarMeN Laboratory, 69600 Oullins, France
4. Univ. Grenoble Alpes, Inserm, UA7 Strobe, 38000 Grenoble, France
5. Hospices Civils de Lyon, 69677 Bron, France
6. IRMB, Univ. Montpellier, INSERM, CHU Montpellier, 34295 Montpellier, France
7. Department of Radiology and Department of Bioengineering, University of Pennsylvania, Philadelphia, Pennsylvania 19104, United States
8. Hôpital Edouard Herriot, 69003 Lyon, France
9. Cell Therapy and Engineering Unit, EFS Rhône Alpes, 38330 Saint Ismier, France
10. Univ. Grenoble Alpes, Translational Innovation in Medicine & Complexity, UMR552, 38700 La Tronche, France
11. Univ. Grenoble-Alpes, Département de Pharmacochimie Moléculaire UMR 5063, 38400 Grenoble, France
12. CHU de Grenoble-Alpes, Institut de Biologie et Pathologie, 38700 La Tronche, France
13. CHU Grenoble Alpes, Stroke Unit, Department of Neurology, 38043 Grenoble, France

‡Equal contribution

* Correspondence: rachel.auzely@cermav.cnrs.fr, marlene.wiart@univ-lyon1.fr

Table of Contents

1. Synthesis of *N*-(2-aminoethyl)-3-acetamido-2,4,6-triiodobenzamide (AcTIB-NH_2)
2. ^{1}H NMR spectra of AcTIB-NHBoc, AcTIB-NH_2, HA-TIB-Fru and HA-TIB-PBA derivatives
3. Cytotoxicity (MTT) assay of individual solutions of HA derivatives incubated with hASCs
4. Rheological analysis of the HA-ref hydrogel in PBS at pH 7.4
5. *In vitro* imaging of the HA-I hydrogel with SKES-CT
6. Volumes of HA-I hydrogel calculated using the reconstructed 3D images obtained by SKES-CT imaging in the knees of osteoarthritic mice in the first 72 h post-injection.
7. *Ex vivo* X-ray phase contrast tomography (XPCT) imaging.
8. Analysis of correlations between the HA-I hydrogel volume evaluated by SKES-CT imaging and its therapeutic effect

1. Synthesis of *N*-(2-aminoethyl)-3-acetamido-2,4,6-triiodobenzamide (AcTIB-NH_2)

3-Acetamido-2,4,6-triodobenzoic acid bis(2-hydroxyethyl)-ammonium salt, (0.2 g, 0.3 mmol) and HATU (0.229 g, 0.60 mmol) were dissolved at room temperature in 50 mL of anhydrous *N*,*N*-dimethylformamide. After stirring for 10 min under nitrogen, *N*-Boc-ethylenediamine (0.096 g, 0.6 mmol) and diisopropylethylamine (0.234 g, 0.0 mmol) were added and the reaction medium was stirred overnight at room temperature under nitrogen. After evaporation of the solvent under reduced pressure, the crude product was dissolved by addition of ethyl acetate. The organic phase was washed thoroughly with 0.5 M HCl, aqueous saturated $NaHCO_3$, and brine, dried over anhydrous sodium sulphate, filtered through filter paper and finally, concentrated by rotary evaporation. *N*-Boc-(2-aminoethyl)-3-acetamido-2,4,6-triiodobenzamide **(AcTIB-NHBoc, Figure S1)** was obtained as a white powder in 85% yield. The structural integrity and purity of the crude product was confirmed by ^{1}H NMR spectroscopy (**Figure S2**).

AcTIB-NHBoc (0.180 g, 0.26 mmol) was then dissolved in a mixture dichloromethane/methanol (1 mL, 9/1, v/v) and excess TFA (5 mL, 65 mmol) was added. After stirring for 4 h at room temperature, the solvent and TFA are removed by rotary evaporation to yield AcTIB-NH_2.

^{1}H NMR (400 MHz, DMSO-d6+D_2O, 25° C) of AcTIB-NH_2: δ_H (ppm) 9.94 (1H, H_{Ar}), 3.44 (2H, CH_2NHCO), 2.99 (CH_2-NH2), 2.02 (3H, CH_3CO).

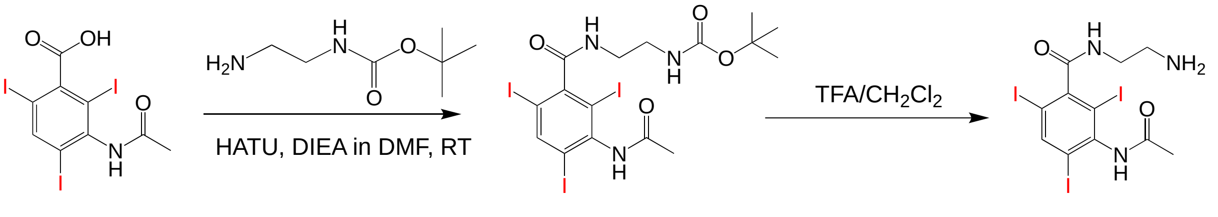

AcTBI AcTBI-NHBoc AcTBI-NH_2

Figure S1. Reaction conditions for the synthesis of AcTIB-NH_2.

2. ^{1}H NMR spectra of AcTIB-NHBoc, AcTIB-NH2, HA-TIB-Fru and HA-TIB-PBA derivatives.

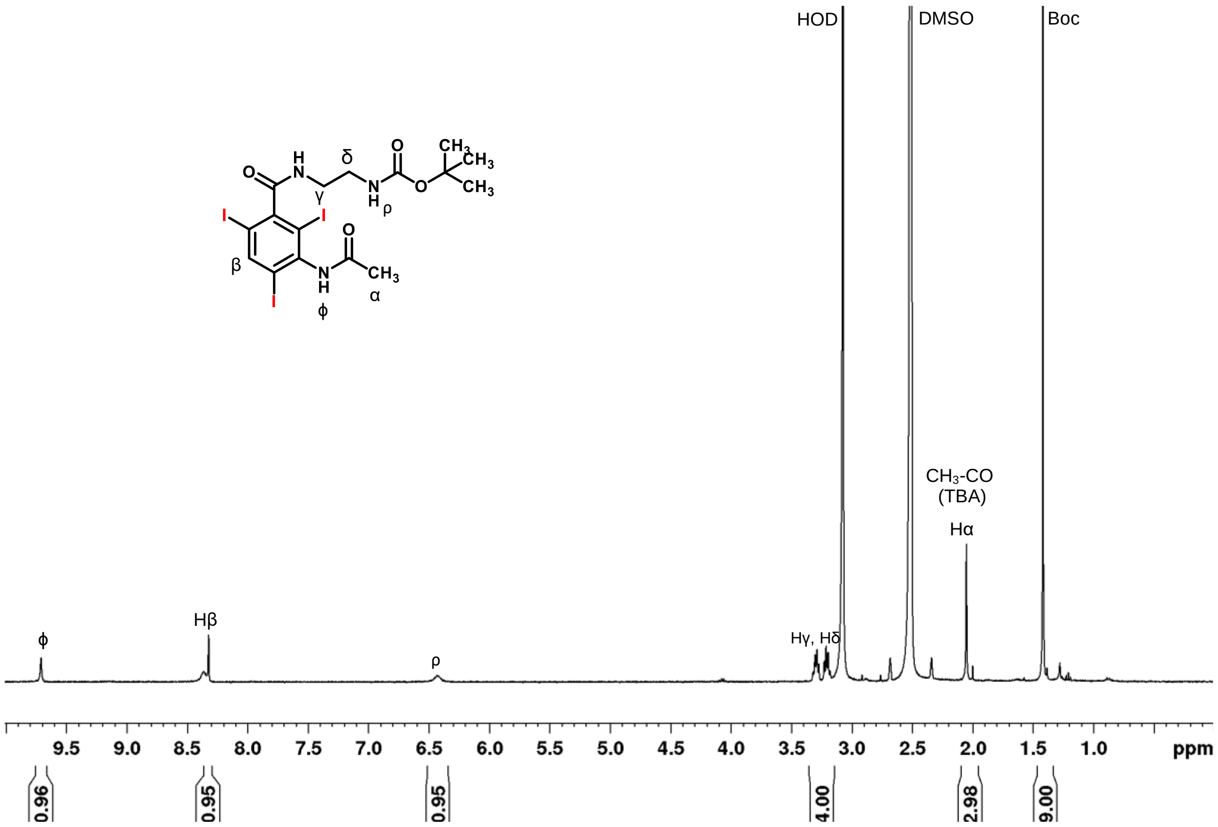

Figure S2. ^{1}H NMR spectrum (400 MHz, DMSO-d6, 6 mg/mL, 25° C) of *N*-Boc-(2-aminoethyl)-3-acetamido-2,4,6-triiodobenzamide (AcTIB-NHBoc).

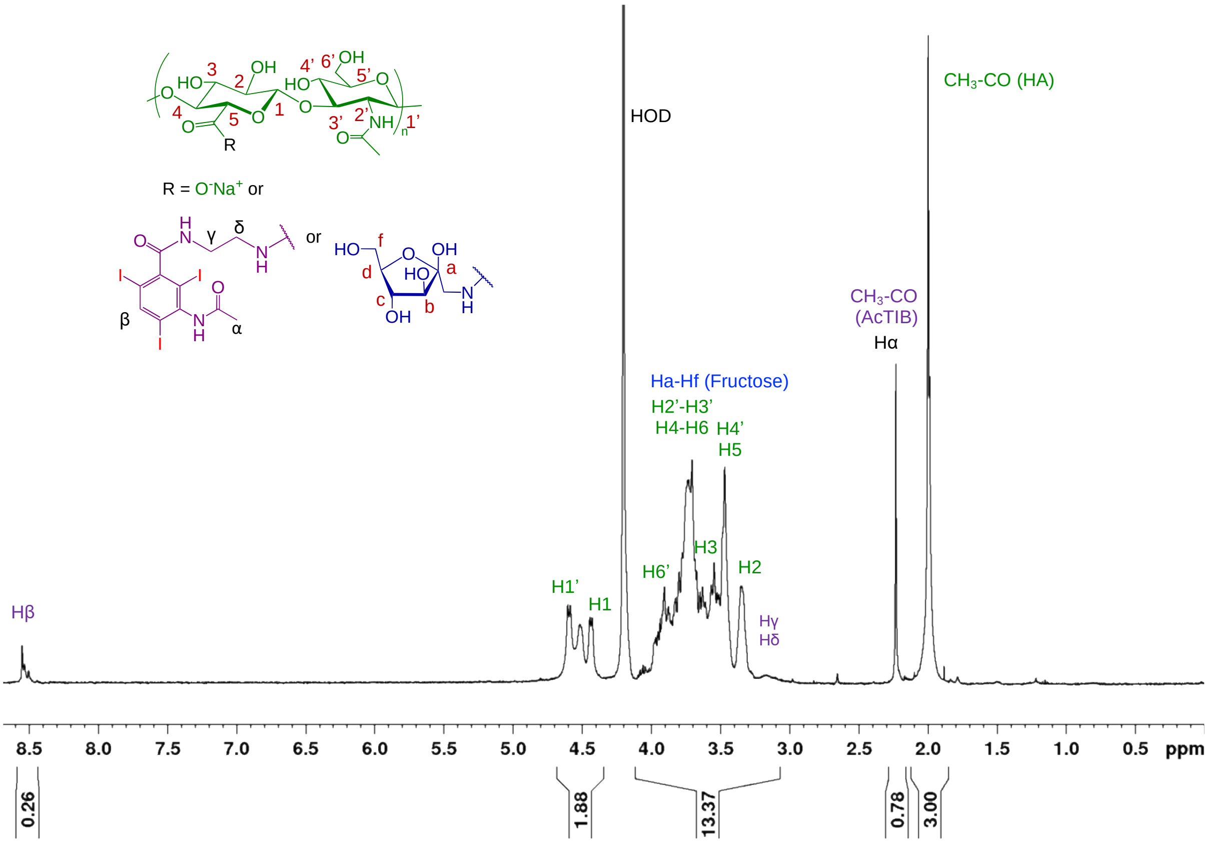

Figure S3. ^{1}H NMR spectrum (400 MHz, D_2O, 6 mg/mL, 80° C) of HA-TIB-Fru.

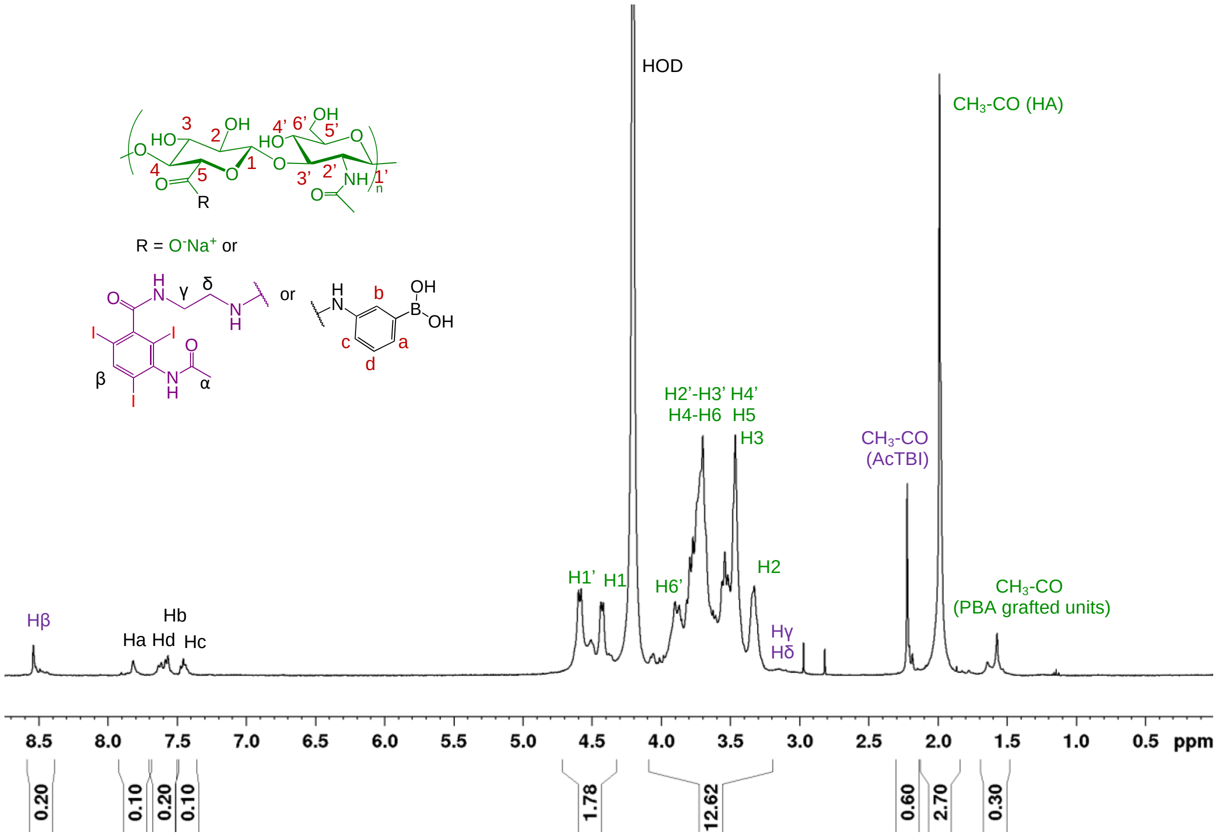

Figure S4. ^{1}H NMR spectrum (400 MHz, D_2O, 6 mg/mL, 80° C) of HA-TIB-PBA.

3. Cytotoxicity (MTT) assay of individual solutions of HA derivatives incubated with hASCs

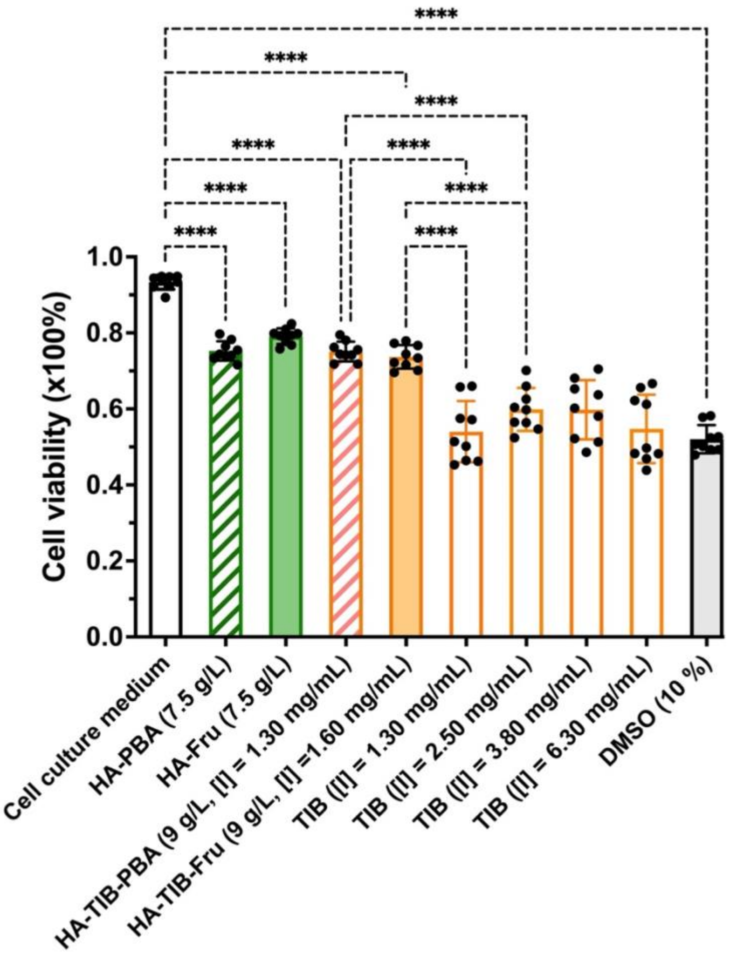

Figure S5. MTT assay of hASCs cultivated for 72 h at physiological conditions (37° C – 5% CO_2) on individual solutions of HA-PBA, HA-Fru, HA-TIB-PBA, HA-TIB-Fru in the cell culture medium (MEM-α supplemented with 1% lysat plaquet and 3% heparin 5 000 U/mL) and a dose-effect study of the iodine commercial contrast agent (3-acetamido-2,4,6-triodobenzoic acid bis(2-hydroxyethyl)-ammonium salt) solutions ([I] = 1.30 mg/mL to 6.30 mg/mL) prepared in 10% DMSO + 90 % cell culture medium. Experiments were repeated with 3 different hASCs from 3 different patient donors in triplicate for each cell batch and for cell culture period. Cell viability results are obtained by UV Visible titrations at 570 nm and represented as mean ± standard deviation. Statistical analysis used the one-way analysis of variance (ANOVA). **** $p < 0.0001$.

4. Rheological behavior of the HA-ref hydrogel in PBS, pH 7.4

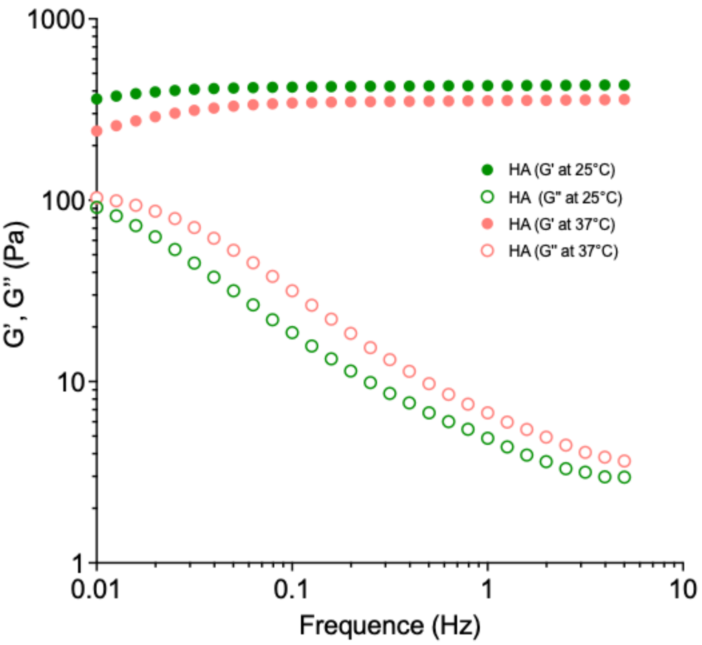

Figure S6. Dynamic rheological behavior of the HA-Fru/HA-PBA hydrogel (PBA/Fru molar ratio of 1) prepared from HA-PBA and HA-Fru in PBS, pH 7.4 (C_p = 12 g/L). Frequency dependence of the storage modulus (G', filled symbols) and loss modulus (G'', empty symbols) measured with 5% strain at 25° C and 37° C.

5. *In vitro* imaging of the HA-I hydrogel with SKES-CT

The figure below illustrates multicolor imaging with SKES-CT of the HA-I hydrogel containing hASCs labeled with gold nanoparticles (AuNPs).

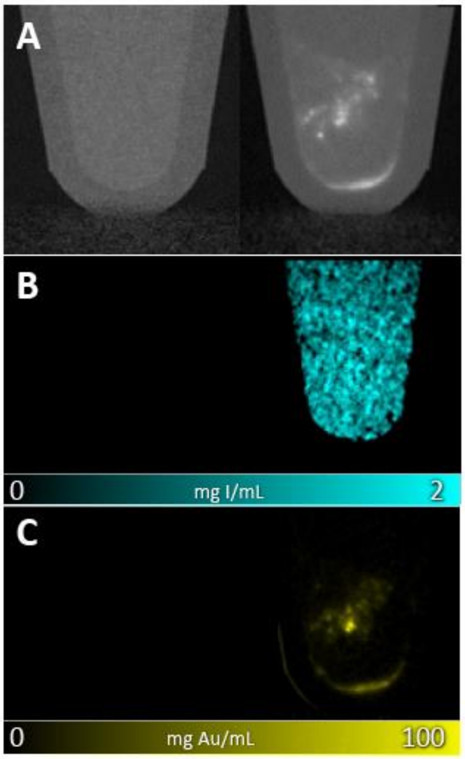

Figure S7. A) Attenuation images of tubes containing an agarose hydrogel as a control tube (left) and gold-labeled hASCs encapsulated in the HA-I hydrogel (right). Iodine and gold (and therefore hydrogel and hASCs) cannot be distinguished on these conventional images (representative single slice from 3D data set). B) Iodine concentration maps highlighting hydrogel distribution. C) Gold concentration maps of the tubes highlighting AuNPs-labeled hASCs distribution. The tube at the right contains 1.25×10^5AuNPs-labeled hASCs encapsulated in the HA-I hydrogel. Cells were labeled following a procedure described previously [1].

6. Volumes of HA-I hydrogel calculated using the reconstructed 3D images obtained by SKES-CT imaging in the knees of osteoarthritic mice in the first 72 h post-injection.

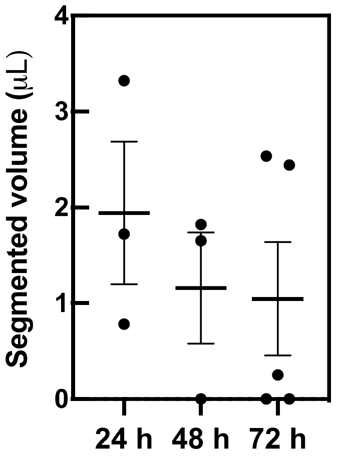

Figure S8. Analysis of the volume of HA-I hydrogel in knee joints at 24h, 48h and 72h post-hydrogel administration (mean ± SEM).

7. *Ex vivo* X-ray phase contrast tomography (XPCT) imaging.

A last synchrotron-base phase contrast X-ray imaging experiment was performed at high resolution. We used a propagation-based experimental set-up with 3.5m sample to detector distance, an optics providing an isotropic voxel size of 3 µm and a beam energy of 26 keV (pink beam with a banwidth of approx. 1keV). This technique has the great advantage to be the simplest experimental set-up allowing to provide phase contrast images of a sample [2]. The figure S8 shows a sagittal view of one mouse knee with a contrast window to show soft tissues. Articular cartilage can be detected on that knee and the hydrogel (indicated by blue arrow) is still be present after 3 days.

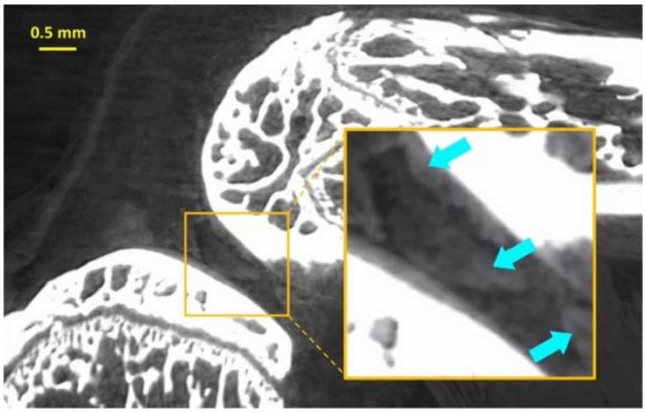

Figure S9. Imaging of the injected HA-I hydrogels with X-ray phase computed tomography (XPCT) in the osteoarthritic mouse knee. The hydrogel can be identified in the joint capsule thanks to the iodine labeling (indicated with the blue arrows and available in Video S2).

8. Analysis of correlations between the HA-I hydrogel volume evaluated by SKES-CT imaging and its therapeutic effect

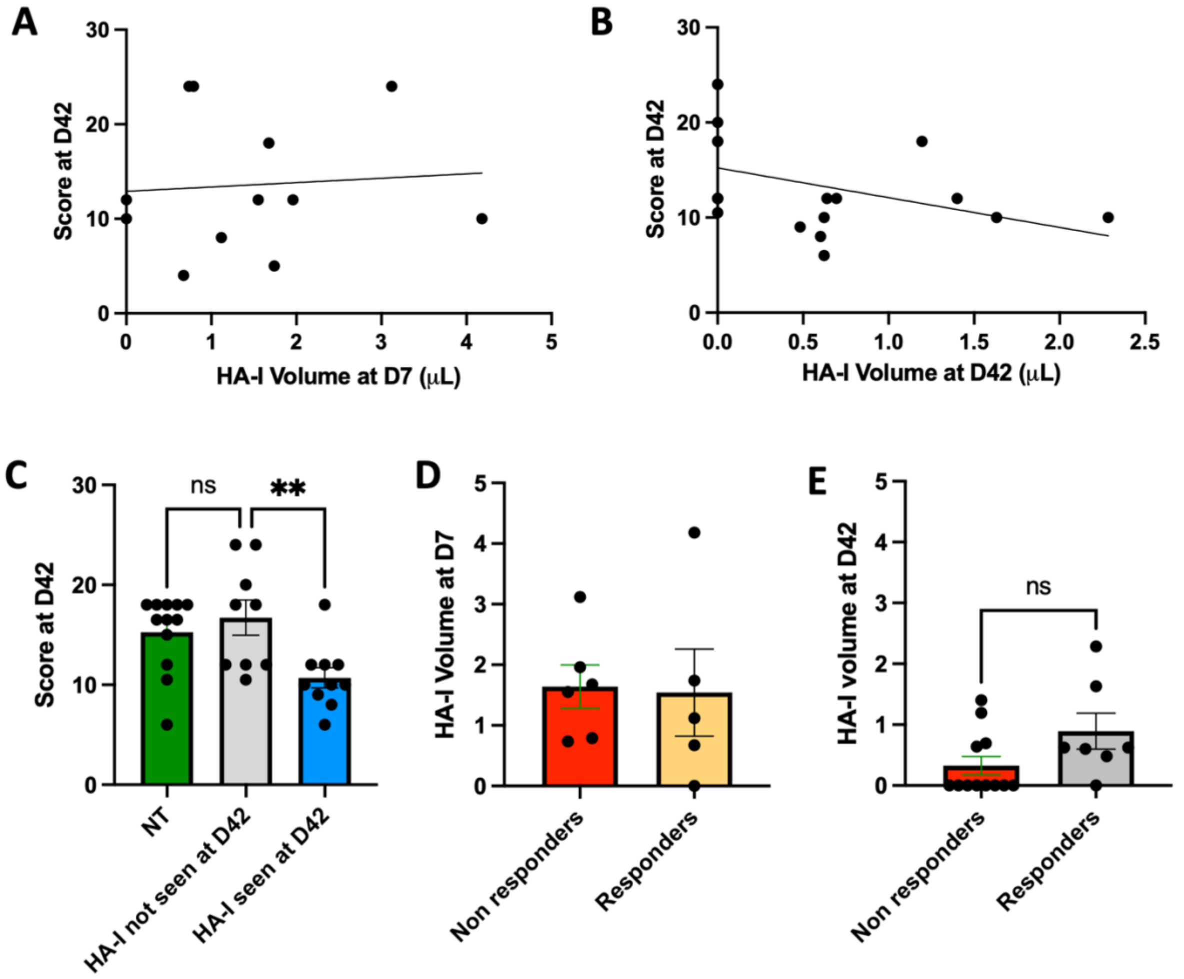

Figure S10. Added value of imaging for predicting therapeutic outcome. A) Correlations between histological score (medial tibial plateau) and hydrogel volume at day 7; B) Correlations between histological score and hydrogel volume at day 42; C) Histological scores in the subgroup of mice in which the hydrogel was still seen at D42 compared to non-treated animals and to the subgroup of mice in which the hydrogel was not seen at D42; D) Volumes of hydrogel at day 7 when mice are stratified as responders and non-responders to treatment; E) Volumes of hydrogel at day 42 when mice are stratified as responders and non-responders to treatment. Analysis of the differences between histological scores at D42 was performed using the Mann–Whitney *U*-test. * $p < 0.05$; ** $p < 0.01$; ns: not significant.

References

[1] Cuccione E, Chhour P, Si-Mohamed S, Dumot C, Kim J, Hubert V, et al. Multicolor spectral photon counting CT monitors and quantifies therapeutic cells and their encapsulating scaffold in a model of brain damage. Nanotheranostics. 2020; 4: 129-41

[2] Quenot L, Bohic S, Brun E. X-ray phase contrast imaging from synchrotron to conventional sources: A review of the existing techniques for biological applications. Appl Sci. 2022; 12:9539.
